# Optimal Treatment Strategies in the Context of ‘Treatment for Prevention’ against HIV-1 in Resource-Poor Settings

**DOI:** 10.1371/journal.pcbi.1004200

**Published:** 2015-04-30

**Authors:** Sulav Duwal, Stefanie Winkelmann, Christof Schütte, Max von Kleist

**Affiliations:** 1 Department of Mathematics and Computer Science, Freie Universität Berlin, Germany; 2 Junior Research Group “Systems Pharmacology & Disease Control”; 3 Zuse Institute Berlin, Germany; Harvard School of Public Health, UNITED STATES

## Abstract

An estimated 2.7 million new HIV-1 infections occurred in 2010. `Treatment-for-prevention’ may strongly prevent HIV-1 transmission. The basic idea is that immediate treatment initiation rapidly decreases virus burden, which reduces the number of transmittable viruses and thereby the probability of infection. However, HIV inevitably develops drug resistance, which leads to virus rebound and nullifies the effect of `treatment-for-prevention’ for the time it remains unrecognized. While timely conducted treatment changes may avert periods of viral rebound, necessary treatment options and diagnostics may be lacking in resource-constrained settings. Within this work, we provide a mathematical platform for comparing different treatment paradigms that can be applied to many medical phenomena. We use this platform to optimize two distinct approaches for the treatment of HIV-1: (i) a diagnostic-guided treatment strategy, based on infrequent and patient-specific diagnostic schedules and (ii) a pro-active strategy that allows treatment adaptation prior to diagnostic ascertainment. Both strategies are compared to current clinical protocols (standard of care and the HPTN052 protocol) in terms of patient health, economic means and reduction in HIV-1 onward transmission exemplarily for South Africa. All therapeutic strategies are assessed using a coarse-grained stochastic model of within-host HIV dynamics and pseudo-codes for solving the respective optimal control problems are provided. Our mathematical model suggests that both optimal strategies (i)-(ii) perform better than the current clinical protocols and no treatment in terms of economic means, life prolongation and reduction of HIV-transmission. The optimal diagnostic-guided strategy suggests rare diagnostics and performs similar to the optimal pro-active strategy. Our results suggest that ‘treatment-for-prevention’ may be further improved using either of the two analyzed treatment paradigms.

## Author Summary

HIV-1 continues to spread globally. Antiviral treatment cannot cure patients, but it slows disease progression and may prevent HIV transmission by decreasing the amount of transmittable viruses in treated individuals. ‘Treatment-for-prevention’ argues for immediate treatment initiation and may reduce transmission by 96% (CI: 73–99%), according to the results of a large clinical study (HPTN052). In order to ensure long-lasting treatment success, early therapy initiation demands more sophisticated treatment strategies & exceeding funds. However, countries facing the highest HIV burden are among the poorest. Within this work, we provide a mathematical framework that allows assessing different treatment paradigms using optimal control theory together with stochastic modelling of within-host viral dynamics and drug resistance development. We use this framework to compute and evaluate two distinct *optimal* long-term treatment strategies for resource-constrained settings: (i) a **diagnostic-guided** and (ii) a **pro-active** treatment **strategy**. The cost of a strategy is evaluated from a national economic perspective, valuating a severe patient health status in terms of an economic loss. The *optimal* strategies are compared with current clinical treatment protocols and **no treatment** in terms of costs, life expectation and reduction of secondary cases. Our simulations indicate that the **pro-active** treatment **strategy** performs comparably to the **diagnostic-guided** treatment **strategy**. Both strategies perform better than current clinical protocols, suggesting directions for improvement.

## Introduction

HIV-1 infection remains one of the major global health challenges with an estimated 33 million infected and a continuing spread [1]. Currently, an efficient vaccine remains to be developed, while at the same time the complete elimination of replication-competent virus within the host can not be achieved due to the persistence of the virus in inducible, latent cellular reservoirs [2, 3], as well as insufficient pharmacological suppression of actively replicating virus in some anatomical/cellular reservoirs [4, 5]. However, the current situation urges for methods that could bring the epidemic to a halt, or possibly end it. Currently, the most promising strategies are based on the use of antiviral drugs:

- Pre-exposure prophylaxis (PrEP) [6–9] aims to protect uninfected individuals ‘at risk’ by decreasing the probability of infection upon virus exposure, e.g. [10]. PrEP may however be too costly to be broadly implemented in resource-poor countries [11].
- Currently, the decision to initiate treatment against HIV is largely guided by CD4+ cell levels [12, 13]. However, the viral load, which is the primary determinant of infectiousness [14, 15], may be very high within the time-window between HIV infection and initiation of treatment. ‘Treatment for prevention’ [16] aims to put infected individuals on therapy as early as possible. This can reduce the infectiousness of a patient by decreasing within-host virus levels, which reduces the amount of transmitted viruses per contact and the probability of infection upon exposure. Analysis of the only completed clinical study to date, HPTN052 [16], estimated that ‘treatment for prevention’ may reduce the number of *linked* HIV-1 transmissions by 96% and the number of *total* HIV-1 transmissions by 89% relative to delayed treatment initiation and subsequently it was nominated as the “breakthrough of the year 2011” by the *Science* magazine [17].

In the aftermath of the HPTN052 trial, the cost-efficacy of ‘treatment for prevention’ was analyzed by many mathematical modeling approaches (reviewed in [18]). One problem is that most of these approaches focused solely on the epidemic level and did not model drug resistance development within the hosts, which indirectly assumes that the efficacy of ‘treatment for prevention’ is constant over time. However, because viral transmission is strongly correlated with viral levels in the transmitting individual [14, 15, 19–21], it is reasonable to assume that also the efficacy of ‘treatment for prevention’ is intimately connected with viral suppression. One major challenge during HIV treatment lies in the virus’ tendency to develop drug resistance [22], which in turn can lead to virus rebound and promote HIV transmission for the time it remains unrecognized. An earlier treatment initiation may thus demand an improved therapeutic strategy, that allows long-term control of virus replication (beyond the typical duration of a clinical trial). While sophisticated patient monitoring and timely treatment changes may allow to minimize windows of unrecognized viral breakthrough, they require significant monetary funds, good infrastructure, diagnostic facilities and the availability of alternative treatment options. Only few of these may be available in resource-constrained countries, where the requirement of resources may strongly dominate the possibility to implement a reasonable ‘treatment for prevention’ strategy. Obviously, scaling ‘treatment for prevention’ requires careful examination of various aspects and a policy maker should strike a proper balance between societal and individual perspectives [23].

This work addresses the scaling of ‘treatment for prevention’ by suggesting *optimal* treatment strategies for the long-term control of HIV within its host (as recommended by [24]). *Optimality* will be defined from a *national economic perspective*, taking into account that a diseased individual implies an economic loss. By considering the national economic perspective, we do not evaluate what *should* be done, but rather what is *already worthwhile*. However, we also evaluate the derived *optimal* strategies from an individual perspective and in terms of their utility in prevention, i.e. whether a strategy prolongs the life of an infected person and whether the risk of HIV onward transmission is reduced.

We hereby focus on two distinct approaches to handle treatment decisions: The first assumes that treatment decisions (i.e. when to change therapy) are made on an *individual* basis, guided by infrequent diagnostics (referred to as **diagnostic-guided strategy**). This represents a medical scenario in which a treating physician decides based on the diagnosed status of the patient that he encounters. The second approach suggests pro-active treatment decisions (referred to as **pro-active strategy**), i.e. does not require diagnostic ascertainment of the patients’ disease status. The two approaches are modeled and solved by two distinct mathematical frameworks. The former is addressed using the recently developed framework of ‘Markov Decision Processes with Rare State Observations’ [25]: For each disease state, it computes the optimal treatment and the next time of medical diagnostics, minimizing viral burden as well as treatment- and diagnostic costs. The latter approach (the **pro-active strategy**) is modeled as an open-loop switched system, where the decision to change the treatment depends on the initial disease state of the patient and the anticipated, (treatment-)induced stochastic dynamics up to some time *t*. The later strategy allows to switch treatment *before* drug resistance is detectable in the individual (pro-active) and may be easier to implement in resource-constrained settings, where poor infrastructure and the costs of diagnostics limit their applicability. By assessing these two distinct frameworks side-by-side, we can rigorously evaluate the different treatment paradigms in terms of their optimality. Algorithms to solve these problems were developed and are stated in the supplementary materials.

Several other groups have suggested *optimal* [26–28] or *sub-optimal* [29, 30] treatment strategies to mitigate drug resistance in HIV-1. All authors treated the underlying system deterministically, which fails to capture the intrinsic stochastic nature of HIV drug resistance development [31] and the time-scales on which drug resistance develops. None of the previous work focused on HIV prevention, and neither work questioned the analyzed treatment philosophy, either focusing on pro-active treatment switching strategies [26–28, 30], *or* diagnostic-driven strategies [29]. In contrast, we used a stochastic model of HIV long-term dynamics after drug application [25] to more realistically capture the underlying dynamics. Also, we evaluate different assumptions for the controllability of the disease dynamics, by evaluating the two different optimal control frameworks, which allows for an objective assessment of alternative treatment philosophies.

The manuscript will be organized as follows: We will extend- and parameterize the model introduced in [25] for our needs. After recapitulating essential theory for the **diagnostic-guided strategy**, we introduce the mathematical concepts behind the **pro-active strategy**, solve both optimal control problems and evaluate them with respect to monetary costs, patient survival and reduction of onward transmission. All algorithms that we developed to solve the optimal control problems will be provided in the S1 and S2 Text for the interested reader.

## Materials and Methods

Within this work, we investigate optimal treatment strategies *in silico* by formulating- and solving two optimal control problems referred to as the optimal **diagnostic-guided strategy** and the optimal **pro-active strategy**. In general, an optimal control problem requires a mathematical model of the controlled process and a performance- or cost criterion. Likewise, our problem will be broken down into these ingredients.

### Model of Controlled HIV Dynamics

**Fig 1:**
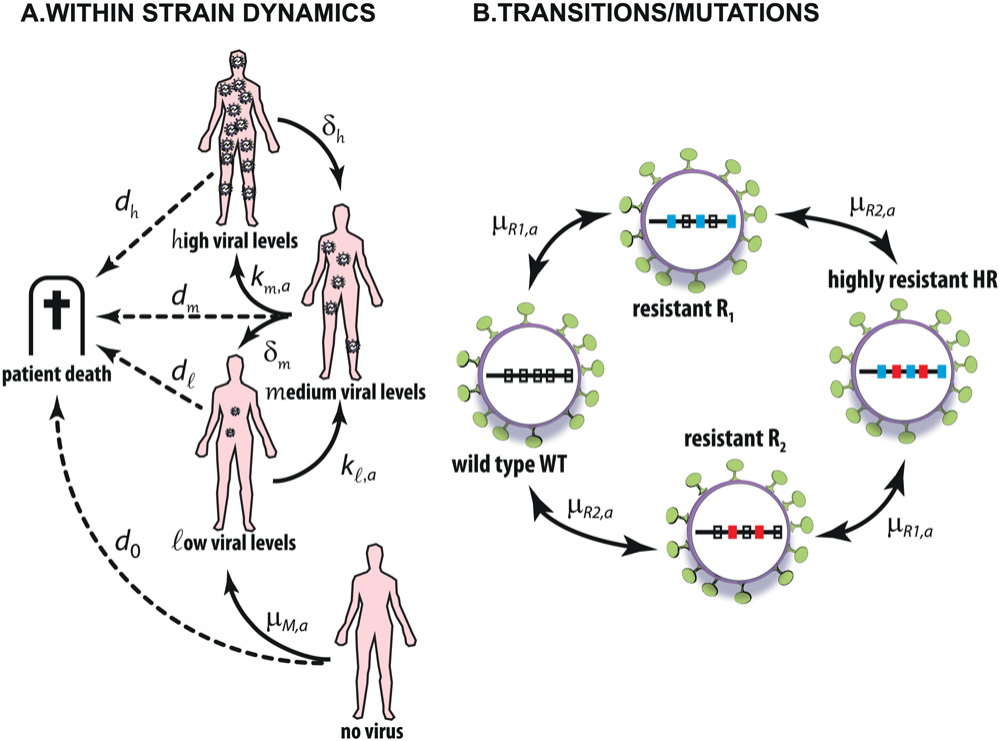
Simplified HIV Model. A: Transitions between copy number states *n*_*C*_. B: Transitions in between viral strains *M*.

**Fig 2:**
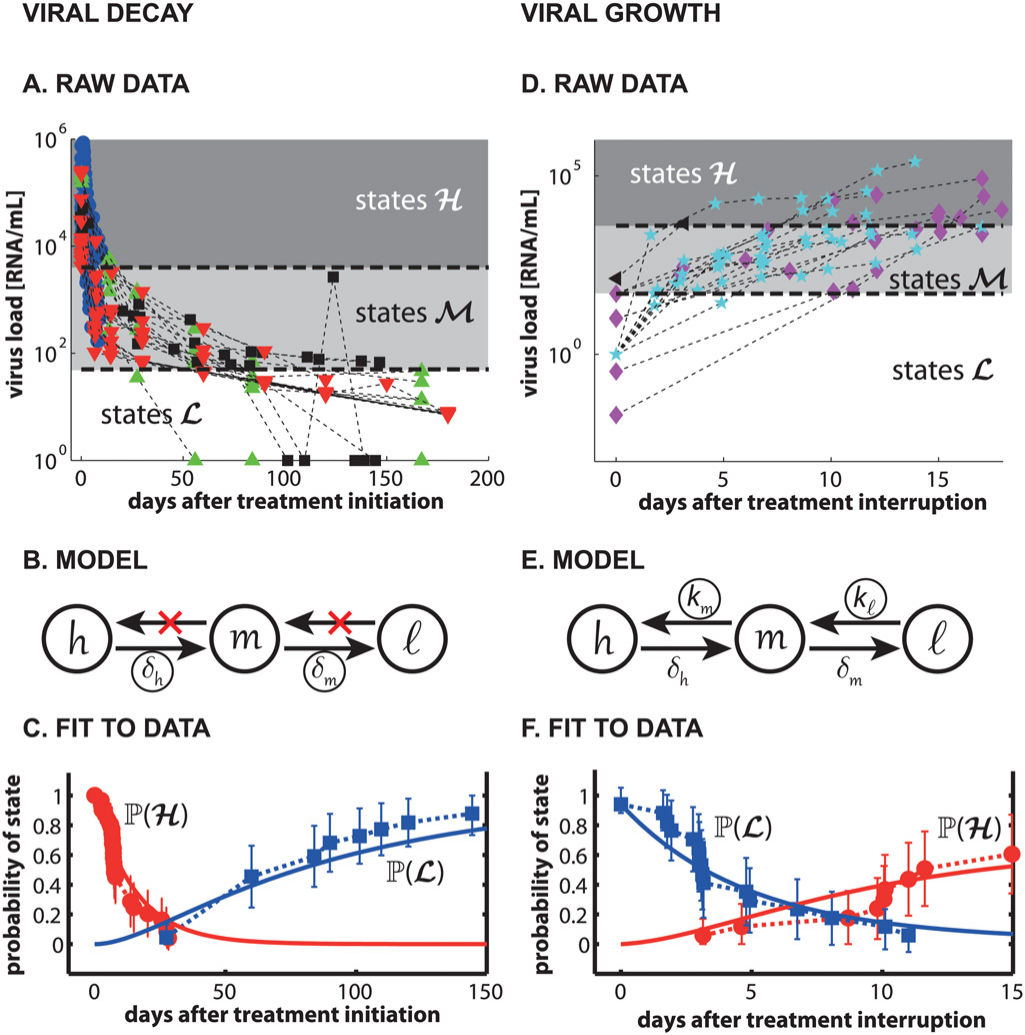
(Short-term) viral dynamics. Left panels (A-C): Viral decay. Right panels (D-F): Viral growth. **A:** Data used for estimating viral decay parameters *δ*_*h*_, *δ*_*m*_. Blue circles indicate viral decay profiles from [41], green upward pointing triangles denote data from [42], black squares denote data from [43] and red downward pointing triangles denote data from [4]. Horizontal dashed lines and background shading indicates the assignment of the depicted data to the sets ℋ (> 4000 viral RNA/mL)), ℳ and ℒ (≤ 50 viral RNA/mL) of our model. **B:** When assuming 100% effective treatment (*η* = 1), the model shown in panel B is derived. This model is used to identify decay parameters *δ*_*h*_ and *δ*_*m*_ (circled parameters in panel B). **C:** Data-derived (error bars, dashed lines) and predicted (solid lines) probabilities of states ℋ and ℒ using the model in panel B with estimated parameters *δ*_*h*_ and *δ*_*m*_. **D:** Data from treatment interruption trials [43–45] used for estimating viral growth parameters *k*_ℓ,∅_, *k*_*m*,∅_. Magenta diamonds indicate viral rebound profiles from [44], cyan pentagrams indicate data from [45] and black left-pointing triangles indicate data from [43]. Horizontal dashed lines and background shading indicates the assignment of the depicted viral growth data to the sets ℋ, ℳ and ℒ. **E:** We assumed the absence of treatment (*η* = 0), such that the model shown in panel E is sufficient to decribe the data and allows identifying growth parameters *k*_ℓ,∅_ and *k*_*m*,∅_ (circled parameters in panel E). **F:** Data-derived (error bars, dashed lines) and predicted (solid lines) probabilities of states ℋ and ℒ using the model in panel E with *δ*_*h*_ and *δ*_*m*_ and estimated parameters *k*_ℓ,∅_ and *k*_*m*,∅_. The parameter estimation procedure is exemplified in the *Material and Methods* section.

**Fig 3:**
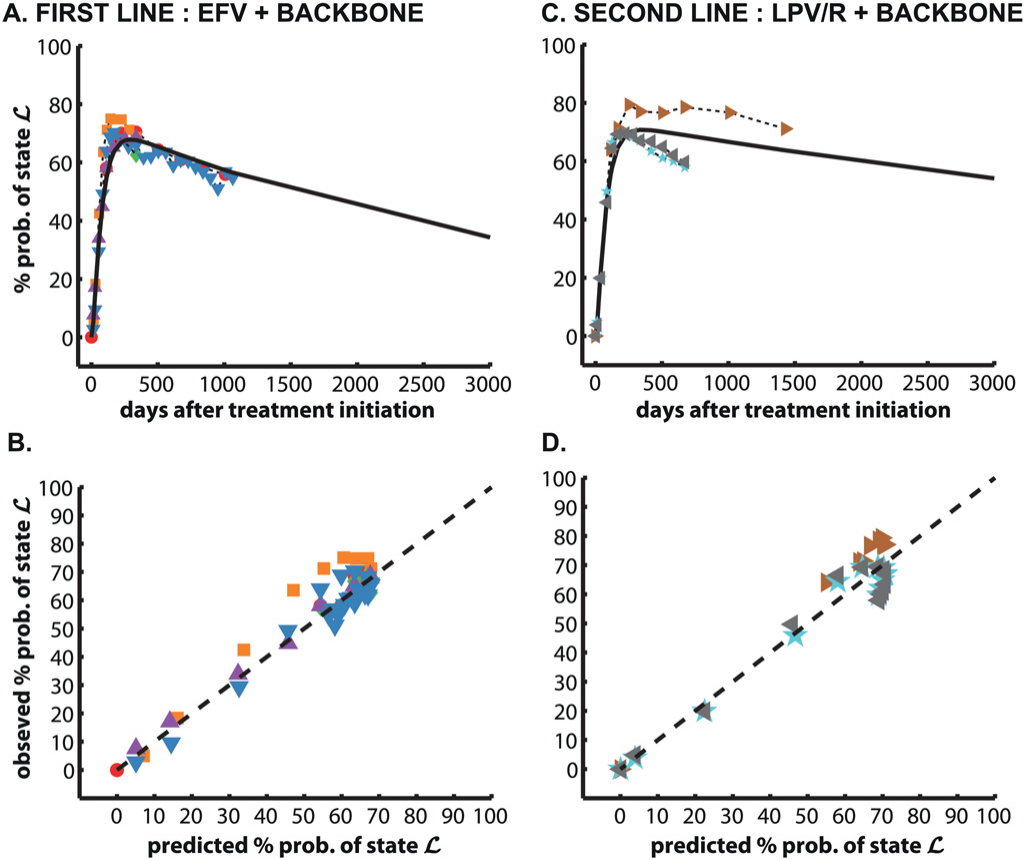
Long-term viral suppression. Long-term data was used to estimate clinical drug efficacy *η*(*a*_1_, {WT, R2}), *η*(*a*_2_, {WT, R1}) and rates of drug resistance emergence *μ*_R1,∅_, *μ*_R2,∅_, using the model depicted in Fig 1, after parameters for viral growth and decay were estimated from data in Fig 2. **A:** Predicted (solid black line) and clinically observed probability of viral suppression (states ℒ; ≤ 50 viral RNA/mL) after treatment with efavirenz (EFV) based HAART (first line therapy). Clinical data was derived from [46] (red dots), [47] (orange squares), [48] (green diamonds), [49] (magenta upward pointing triangles) and [50] (blue downward pointing triangles). In all studies, the NRTI backbone consisted of 3TC + AZT. **B:** Goodness-of-fit plot for first line therapy. **C:** Predicted (solid black line) and clinically observed probability of viral suppression (states ℒ; ≤ 50 viral RNA/mL) after treatment with ritonavir boosted lopinavir (LPV/r) based HAART (second line therapy). Clinical data was derived from [51] (brown right-pointing triangles), [52] (cyan pentagrams) and [52] (grey left-pointing triangles). In all studies, the NRTI backbone consisted of a deoxycytidine analog + abacavir or tenofovir or stavudine, reflecting clinical practice (the exact choice of the backbone may depend on prior exposure [13]). **D:** Goodness-of-fit plot for second line therapy.

The two addressed optimal control approaches share an identical model (Fig 1) that reflects the short-term dynamics of viral decay- and rebound (Fig 2), as well as the stochastic HIV long-term dynamics after drug application, see Fig 3. Within this work, we put a focus on viral kinetics and will only indirectly relate to the patient’s health. This is because we are interested in ‘treatment for prevention’ and particularly its efficacy in decreasing onward transmission, which is correlated with the viral load [19–21] and not necessarily with the immune status of the HIV infected patient.

#### State space

HIV can be successfully suppressed if drug resistance does not develop. Thus, any model that aims to represent the long-term HIV dynamics upon treatment should include drug resistance development. The process of drug resistance development denotes an intrinsically stochastic process, which is determined by random mutation events (point mutations, recombinations). Long term HIV-dynamics in the context of drug treatment may therefore be dominated by these intrinsically stochastic events [31], necessitating stochastic modeling approaches [32–34]. The fundamental evolution equation for intrinsically stochastic kinetics is the chemical master equation (CME). Each state described by the CME comprises a combination of discrete numbers of individuals of the respective species (e.g. viral strains), resulting in state space dimensions ℕ_0_×ℕ_0_×…×ℕ_0_, i.e. [35, 36]. A major mathematical drawback is the fact that the CME cannot be solved directly due to this complexity. Therefore, a modeler can either approximate the solution of the CME by Monte-Carlo schemes [37], aim at hybrid approaches [38–40], which can yield particular characteristics of the CME, or perform a state space reduction (lumping). In this manuscript, we adapt a model [25] that relates to the latter approach. For this model we can solve the coarse-grained CME directly when computing optimal control strategies.

In brief, the HIV model contains four lumped viral copy number states for each of the four virus strains. The set of states 𝒮 thus has dimension 4^4^ = 256 states + 1 [patient death]: If the respective virus type is absent, we denote the respective state by 0, if it is present in low copy numbers, i.e., for < 50 virus copies/mL blood (detection limit of assays used in the clinic), the respective state is denoted by ℓ, for medium copy numbers between 50 and 4000 virus copies/mL blood we denote the lumped states by *m* and for high copy numbers with more than 4000 virus copies/mL blood, it is *h*. This coarse graining is in line with the levels of virus produced in the distinct cellular reservoirs of HIV, see e.g. [34]. The following four viral strains *M* are considered: a strain WT (wild type) that is susceptible to all treatment lines, a strain R1 which is susceptible to a second treatment line, but unaffected by (resistant to) the first treatment line, a strain R2 that is susceptible to the first treatment line, but unaffected by the second, and a highly resistant strain HR, which is resistant to all treatments. In order to describe a virologic state *x* we choose a compact vector notation of the form

$$\begin{array}{c} x=[n_{C}\left(\mathrm{WT}\right),\;n_{C}\left(R1\right),\;n_{C}\left(R2\right),\;n_{C}\left(\mathrm{HR}\right)], \end{array}$$

where *n*_*C*_ ∈ {0, ℓ ,*m*, *h*} denotes the viral copy number of each viral strain WT, R1, R2 or HR. For example, the state x=[ℓ,m,0,ℓ] describes the situation of a ℓow number of wild type strains, a *m*edium number of R1-mutants, the absence of R2-mutants, and a ℓow number of highly resistant viruses. Mutations from one strain to another can give rise to novel viral populations, as shown in Fig 1.

#### Control actions

The actions describe ‘what the controller can do to influence the system’. In terms of HIV therapy, a physician can e.g. choose what treatment(-line) to apply and when to change it. In resource-constrained settings, only few treatment lines are available. In the case of South Africa these may include a first- and a second-line therapy [13]. Taking these considerations in account, we consider two distinct treatment lines (actions) *a*_1_,*a*_2_ ∈ 𝒜. Each action *a* ∈ 𝒜 induces unique disease dynamics, related to a unique *Markov Jump Process* that is entirely determined by its infinitesimal generator *L*_*a*_. The entry *L*_*a*_[*x*,*y*] ≥ 0 represents the rate of transition from state *y* ∈ 𝒮 to state *x* ∈ 𝒮, *y* ≠ *x*, given an action *a* and it holds that *L*_*a*_[*y*,*y*] = −∑_*x* ≠ *y*_ *L*_*a*_[*x*,*y*]. We define a probability space Ω and let *p* ∈ Ω denote a probability distribution vector on the state space 𝒮 with the entry *p*[*x*](*t*) referring to the probability of being in the state *x* ∈ 𝒮 at time *t*, i.e.

$$\begin{array}{c} p\left[x\right]\left(t\right):=\mathbb{P}\left(X_{t}=x\right), \end{array}$$ (1)

where ℙ is the probability measure. Obviously, the number of components of a probability vector *p* is equal to |𝒮|. For a given action *a* ∈ 𝒜, the dynamics of the probability vector are given by

$$\begin{array}{c} \frac{dp(t)}{dt}=L_{a}\cdot p\left(t\right) \end{array}$$ (2)

The above equation is known as the *Master Equation*. We introduce the transpose of the transition matrix on 𝒮 for some time lag *τ* and action *a*

$$\begin{array}{c} T_{a,\tau }:{\mathbb{R}}^{\left|𝒮\right|}\mapsto {\mathbb{R}}^{\left|𝒮\right|},\;T_{a,\tau }p:=e^{L_{a}\cdot \tau }p, \end{array}$$ (3)

where **e** denotes the matrix exponential. The component *T*_*a*,*τ*_[*x*,*y*] refers to the transition probability from state *y* to state *x* for a time lapse *τ* under the application of action *a* and will be used later in the cost functionals of the closed-loop optimal control problem (**diagnostic-guided strategy**) and the open-loop optimal control problem (**pro-active strategy**).

#### Generator entries

The distinct treatments *a* ∈ 𝒜 are related to distinct generators *L*_*a*_ of our HIV-model. The basic transitions between copy number states for each viral strain *M*, *n*_*C*_(*M*), are shown in Fig 1 and exemplified for the highly resistant strain HR below.

$$\begin{array}{c} \left[*,\;*,\;*,\;\ell ]\overset{\overset{k_{\ell ,a}}{⟶}}{\underset{\delta_{m}}{⟵}}[*,\;*,\;*,\;m],\;[*,\;*,\;*,\;m]\overset{\overset{k_{m,a}}{⟶}}{\underset{\delta_{h}}{⟵}}[*,\;*,\;*,\;h\right] \end{array}$$ (4)

$$\begin{array}{c} {}[*,\;*,\;*,\;h]\overset{d_{h}}{⟶}✠,[*,\;*,\;*,\;m]\overset{d_{m}}{⟶}✠,[*,\;*,\;*,\;\ell ]\overset{d_{\ell }}{⟶}✠, \end{array}$$ (5)

where * indicates an arbitrary number of the respective virus strain (WT, R1 and R2 in the example above). The parameters *k*_ℓ,*a*_ and *k*_*m*,*a*_ denote the reaction propensities of going from copy number ℓ to copy number *m* and from copy number *m* to copy number *h* respectively (viral growth), which are decreased depending on the treatment *a* ∈ {*a*_1_, *a*_2_} because treatment essentially suppresses viral growth. The parameters *δ*_*m*_ and *δ*_*h*_ denote the reaction propensities for going from copy number *m* to copy number ℓ and from copy number *h* to copy number *m* respectively (virus elimination). The parameters *d*_*h*_ > *d*_*m*_ > *d*_ℓ_ denote the propensity for the death of the patient. We assume that high viral burden (states *h* and *m* respectively) increases the risk of death, whereas *d*_ℓ_ equals the propensity for “natural death”. The propensity for death was computed according to *d* = 1/(residual life expectancy), and is exemplified in [25].

The considered transitions between viral strains *M* are depicted in Fig 1. Specifically, transitions between viral strains generate a ℓow number of viral particles from either a *m*edium or *h*igh number of viruses belonging to a distinct strain. Note, that transitions between viral strains may involve several distinct point mutations (indicated by blue and red bars in Fig 1B). Exemplified for the wild type strain WT those are:

$$\begin{array}{ccc} {}[\;h,\;0,\;*,\;*\;]\overset{\mu_{R1,a}}{⟶} & [\;h,\;\ell ,\;*,\;*\;],\; & \left[\;m,\;0,\;*,\;*\;]\overset{\mu_{R1,a}}{⟶}[\;m,\;\ell ,\;*,\;*\;\right] \end{array}$$ (6)

$$\begin{array}{c} \left[\;h,\;*,\;0,\;*\;]\overset{\mu_{R2,a}}{⟶}[\;h,\;*,\;\ell ,\;*\;],\;[\;m,\;*,\;0,\;*\;]\overset{\mu_{R2,a}}{⟶}[\;m,\;*,\;\ell ,\;*\;\right] \end{array}$$ (7)

$$\begin{array}{c} \left[\;0,\;h,\;*,\;*\;]\overset{\mu_{R1,a}}{⟶}[\;\ell ,\;h,\;*,\;*\;],\;[\;0,\;m,\;*,\;*\;]\overset{\mu_{R1,a}}{⟶}[\;\ell ,\;m,\;*,\;*\;\right] \end{array}$$ (8)

$$\begin{array}{c} \left[\;0,\;*,\;h,\;*\;]\overset{\mu_{R2,a}}{⟶}[\;\ell ,\;*,\;h,\;*\;],\;[\;0,\;*,\;m,\;*\;]\overset{\mu_{R2,a}}{⟶}[\;\ell ,\;*,\;m,\;*\;\right] \end{array}$$ (9)

where the first two lines indicate drug resistance *arising* from the wild type strain and the remaining two lines indicate transitions from resistant strains *yielding* the wild type strain. The parameters *μ*_R1,*a*_ and *μ*_R2,*a*_ denote the propensity for the emergence- and disappearance of drug resistance to treatment 1 or 2 (*a*_1_,*a*_2_), respectively, emanating from copy number state *h* or *m*. Note, that we consider only the following transitions: WT ↔ R1, WT ↔ R2, R1 ↔ HR and R2 ↔ HR, which is motivated by the fact that a direct transition from WT ↔ HR is very unlikely, because the genetic distance between the two viral strains is too large to be overcome at once.

The effect of treatments *a*_1_ and *a*_2_ on the viral growth & transition rates is considered in the following way:

$$\begin{array}{c} k_{\ell ,a}=(1-\eta (a,M))k_{\ell ,\emptyset } \end{array}$$ (10)

$$\begin{array}{c} k_{m,a}=(1-\eta (a,M))k_{m,\emptyset } \end{array}$$ (11)

$$\begin{array}{c} \mu_{\tilde{M},a}=(1-\eta (a,M))\mu_{\tilde{M},\emptyset } \end{array}$$ (12)

where *M* ∈ {WT, R1, R2, HR} denotes the strain of the reactant virus. $\tilde{M}\in \{\text{WT},\text{R1},\text{R2},\text{HR}\}$ denotes the event related to a particular drug resistance emergence/disappearance, see Fig 1B. The parameter *η*(*a*,*M*) denotes the efficacy of treatment *a* on the reactant viral strain *M*; i.e. if strain *M* is susceptible to treatment *a* ∈ {*a*_1_, *a*_2_}, then 0 < *η*(*a*,*M*) ≤ 1, and if the viral strain *M* is insusceptible to treatment *a* ∈ {*a*_1_, *a*_2_} then *η*(*a*,*M*) = 0. In the absence of medical intervention *a* = *a*_∅_, *η*(*a*,*M*) = 0. Therefore, the parameters *k*_ℓ,∅_, *k*_*m*,∅_ and $\mu_{\tilde{M},\emptyset }$ denote the growth rates and respective transition rates in copy number states *m* and *h* in the absence of intervention, as shown in Table 1.

**Table 1:** Parameters of the HIV-model.

parameter	value	parameter	value
*k*_ℓ,∅_	0.2027	*k*_*m*,∅_	0.1308
*δ*_*m*_	1.13·10^−2^	*δ*_*h*_	6.62·10^−2^
*d*_ℓ_	9.4·10^−5^	𝕀ℝ(*n*_*C*_ = ℓ)	0.2
*d*_*m*_	2.7·10^−4^	𝕀ℝ(*n*_*C*_ = *m*)	1.85
*d*_*h*_	5.5·10^−4^	𝕀ℝ(*n*_*C*_ = *h*)	13.18
*μ*_R1,∅_	1.739·10^−1^	*μ*_R2,∅_	2.54·10^−2^
*η*(*a*_1_, {WT, R2})	0.9894	*η*(*a*_1_, {R1, HR})	0
*η*(*a*_2_, {WT, R1})	0.9825	*η*(*a*_2_, {R2, HR})	0

Infection risks 𝕀ℝ were derived from data, as explained in S5 Text. Parameters *d*_ℓ_, *d*_*m*_ and *d*_*h*_ were estimated from life-expectation data as explained in [25]. All other parameters were estimated from data shown in Figs 2 and 3 and exemplified in the *Material & Methods* section. All values are given in units [1/day] except *η* [unit less] and 𝕀ℝ [per 100 person-years].

#### Parameter estimation

In order to estimate model parameters, we proceeded in a step-wise approach: We first estimated parameters related to viral decay (*δ*_*h*_, *δ*_*m*_) and then used these estimates in order to estimate parameters related to viral growth in the absence of treatment (*k*_ℓ,∅_, *k*_*m*,∅_), using data from [4, 41–45]. Finally, we used the estimated decay- and growth parameters along with data on the long-term (> 2 years) suppression of HIV-1 in order to estimate parameters related to the drug efficacy (*η*(*a*_1_, {WT, R2}), *η*(*a*_2_, {WT, R1})) and to drug resistance development (*μ*_R1,∅_, *μ*_R2,∅_) [46–52].

Parameters were estimated in MATLAB using *lsqcurvefit* by minimizing the following weighted least squares criteria, with *θ* denoting the set of estimable parameters.

$$\begin{array}{c} \theta^{*}=\underset{\theta }{\mathrm{argmin}}\sum_{i}{\left(\frac{\pi \left[x\right]\left(t_{i}\right)-p\left[x\right]\left(t_{i},\theta \right)}{\omega_{i}}\right)}^{2} \end{array}$$ (13)

where *π*[*x*](*t*_*i*_) denotes the data-derived probability distribution on the model-defined state-space (computed using the *ecdf* function in MATLAB), *p*[*x*](*t*_*i*_,*θ*) defines the solution of Eq (2) for time *t*_*i*_ with parameter set *θ* and *ω*_*i*_ denotes the weight parameter. Parameter estimation was performed 50 times respectively with random start parameters to verify the convergence to globally optimal parameter estimates *θ* ^*^.

##### Viral decay

A total of 311 data points from 31 patients and 4 independent clinical studies were available from [4, 41–43], which accurately assess the dynamics of viral decay after initiation of treatment (see Fig 2A). For the data analyzed, we assumed 100% effective treatment (*η* = 1), as proposed by others who estimated viral decay parameters [41, 53]. The lumped viral model (see Fig 1) then further reduces to the model shown in Fig 2B, which allows to identify decay parameters *δ*_*h*_ and *δ*_*m*_. The data-derived probabilities *π*[*x*](*t*_*i*_) were computed as 1− the cumulative probability to leave set ℋ (> 4000 viral RNA/mL) and the cumulative probability to enter set 𝓛 (≤ 50 viral RNA/mL). Error bars were computed using Green’s formula. In line with the data, we assumed that the initial HIV virologic status is represented by high copy numbers of susceptible virus.

##### Viral growth

A total of 89 data points from 17 patients and 3 treatment interruption trials [43–45], was used to estimate viral growth parameters *k*_ℓ,∅_ and *k*_*m*,∅_. In line with the data, we assumed the absence of treatment (*η* = 0), such that the model shown in Fig 2E is sufficient to describe the data. Data-derived probabilities were computed as 1− the cumulative probability to leave set 𝓛 and the cumulative probability to enter set ℋ, respectively, and error bars were computed using Green’s formula.

##### Drug efficacy and -resistance

Using the full model (Fig 1), we estimated parameters relating to the clinical drug efficacy of both treatment lines *η*(*a*_1_, {WT, R2}) & *η*(*a*_2_, {WT, R1}) and rates of drug resistance emergence *μ*_R1,∅_ and *μ*_R2,∅_.

In analogy with the South African treatment guidelines, we assumed that the first-line therapy consists of efavirenz (EFV) + zidovudine (AZT) + lamivudine (3TC). Long-term studies usually evaluate the probability of viral suppression, which is defined in terms of undetectable virus loads (≤ 50 viral RNA/mL). Translated to our model, this refers to the condition in which all viral mutants are in state ℓ or absent; i.e. [≤ℓ,≤ℓ,≤ℓ,≤ℓ], which we denote by the set of states by 𝓛. Probabilities of viral suppression from 5 clinical studies [46–50] were used for parameter estimation. As a second-line treatment we assumed a ritonavir-boosted lopinavir (LPV/r) based HAART, see [13]. Since the exact choice of the NRTI backbone may depend on the prior exposure of the individual patient, we used data evaluating the long-term efficacy of LPV/r + an NRTI backbone consisting of a deoxycytidine analog + stavudine [51] or abacavir [52] or tenofovir [52].

All model parameters are shown in the Table 1. The original data and model predicted dynamics of viral decay and -rebound are shown in Fig 2 (A: raw viral decay data; B: model to evaluate viral decay; C: model-predicted vs. clinical decay profiles; D: raw viral growth data; E: model to evaluate viral growth; F: model-predicted vs. clinical growth profiles). Data for the long-term control of HIV-1, predicted dynamics and goodness-of-fit are shown in Fig 3A–3D for the two treatment lines (*a*_1_ and *a*_2_). As can be seen in Figs 2 and 3, the model appropriately captures both the short-term viral dynamics, as well as long-term dynamics of viral suppression.

#### Cost assignment

Public health initiatives are often constrained by available funds. The countries with the highest HIV burdened are also among the poorest and financial commitments from donors have stagnated or decreased [54] in recent years. Thus, the requirement of resources may strongly dominate the policy making process in a resource-constrained context. Because of these conditions, we designed the performance criterion from a national economic perspective.

The performance criterion valuates the induced system dynamics and controls, i.e. the viral status of the patient and the costs of treatment. We will consider both the direct costs due to the applied treatments *c*_𝒜_ and indirect costs due to the virologic/health status of a patient *c*_𝒮_. Our analysis will be conducted from a country’s public health-care/monetary perspective. Therefore, the costs related to the different states *c*_𝒮_ will be computed based on the average productivity loss pL(*n*_*C*_) times the average daily monetary contribution of one individual (assessed in terms of the daily per capita GDP), i.e. *c*_𝒮_(*x*) = pL(*x*)·GDP, with $\text{pL}(x)=\underset{n_{C}}{\max}\;\;\text{pL}(n_{C})$, which implies that the total virus load reflects the cost of the individual infection status at any point in time. Death is interpreted in terms of a complete loss in productivity. Furthermore, we take diagnostic costs into account, which applies only in the **diagnostic-guided strategy**, the **standard of care** and the **HPTN052 protocol** (the latter two are modeled for comparison). The cost of diagnostics will be set to a fixed value and closely reflect the cost of a drug resistance test for the **diagnostic-guided strategy** and the cost of a virus load determination in the case of the **standard of care** and the **HPTN052 protocol**.

The integration of momentary/running costs yields the objective function (performance criterion) for the optimal control problem. While performance criteria generally depend on the particular application at hand, we decided to consider expected discounted costs on an infinite time horizon. We chose an infinite time horizon, because HIV treatment does not have a previously known endpoint (i.e. time of death). At the same time, a differentiated weighting of immediate and later costs is reasonable due to an upper limitation of life expectancy and aspects of inflation. Costs arising at time *t* > 0 are thus weighted by a discount factor 0 < *e* ^−*λt*^ < 1. In this regard, the concrete choice of a discount factor *λ* will depend on the presumed annual inflation in the considered setting. For all calculations, we consider the inflation rate in South Africa as a representative of a resource-constrained country with a large HIV burden, see Table 2. The discount factor also guarantees convergence of the cost functional and therefore allows the numerical solution of the optimal control problem.

**Table 2:** Cost parameters for South Africa.

parameter	value	unit	reference
*c*_𝒜_(*a*_1_)	0.3	US$/d	[65]
*c*_𝒜_(*a*_2_)	1.08	US$/d	[65]
*k*_dia_	200	US$	[57, 59]
GDP	6,620	US$/p.p./y	[75]
pL(*n*_*C*_ = ℓ)	0	-	[76]
pL(*n*_*C*_ = *m*)	0.1	-	[76]
pL(*n*_*C*_ = *h*)	0.4	-	[76]
pL(✠)	1	-	-
*λ*	1.47·10^−4^	1/d	^a^

*k*_dia_ refers to the price for a drug resistance test. The GDP refers to the estimation for the year 2013 by the International Monetary Fund [75]. The state costs are defined by $c_{𝒮}(x)=\underset{n_{C}}{\max}\;\text{pL}(n_{C})\cdot \text{GDP}$.

^*a*^ Assuming an annual inflation of 5.4% for South Africa [75].

The costs per unit time comprise both the direct costs due to the applied treatments and indirect costs due to the virologic/health status of a patient. Thus, we can write

$$\begin{array}{c} c\left(x,a\right)=c_{𝒮}\left(x\right)+c_{𝒜}\left(a\right) \end{array}$$ (14)

where *c*_𝒜_:𝒜 ↦ [0, ∞) is the direct cost of action per unit time and *c*_𝒮_:𝒮 ↦ [0, ∞) is the indirect cost produced by the state per unit time with parameters given in Table 2.

We define a cost function

$$\begin{array}{c} C(x,a,\tau ):={𝔼}_{x}^{a}(\int_{0}^{\tau }e^{-\lambda s}c\left(X_{s},a\right)\;ds), \end{array}$$ (15)

which denotes **the expected discounted costs for the time interval (0,*τ*]** when starting in state *x* and choosing an action *a* ∈ 𝒜 for propagation of the stochastic process for the entire interval *τ*. Further, we define the cost vector 𝒦_*a*_ ∈ ℝ^|𝒮|^, where its x^th^ component denotes the direct and indirect cost per unit time for the state *x* ∈ 𝒮 as shown below

$$\begin{array}{c} {𝒦}_{a}\left[x\right]:=c\left(x,a\right), \end{array}$$ (16)

so that it holds that

$$\begin{array}{c} C\left(x,a,\tau \right):={𝒦}_{a}^{'}(\int_{0}^{\tau }e^{-\lambda s}\cdot e^{L_{a}\cdot s}\;ds)\varphi_{x}, \end{array}$$ (17)

where the vector *φ*_*x*_ denotes a point-distribution, i.e. a single realization *X*_*t*_ of the Markov Jump Process. If the initial state is described by an arbitrary distribution *p* on the state space 𝒮, we get

$$\begin{array}{c} C\left(p,a,\tau \right)=\sum_{x\in 𝒮}C\left(x,a,\tau \right)\cdot p\left[x\right], \end{array}$$ (18)

where *p*[*x*] denotes the probability of the x^th^ state.

### Performance Criterion and Bellman Equation

The two optimal control problems that we solve, i.e. the **diagnostic-guided strategy** and the **pro-active strategy**, differ slightly in the underlying assumption on the controllability of the disease dynamics. Both control strategies will be described in the following, defining in each case a control policy, a performance criterion and an optimality equation.

#### Diagnostic-guided strategy (closed-loop optimal control)

In the **diagnostic-guided strategy**, treatment can only be changed after a (costly) diagnostic test has been made to determine the virologic state of the patient (i.e. the drug resistance profile). This would correspond to the typical scenario in which a treating physician makes a *patient-specific* decision. However, instead of considering regular diagnostic intervals, we consider *patient-specific* diagnostic intervals. That is, upon assessing the virologic status of the patient, the physician decides both on a treatment *a* and on a time-lag *τ* until the next diagnosis. This implies that patients, whose viral status is “critical” may be monitored more closely than those whose status is “uncritical”. More precisely, a *policy* for the diagnostic-guided strategy is a function

$$\begin{array}{c} u:𝒮\to 𝒜\times [0,\infty ),\;\;\;x\mapsto u(x)=(a(x),\tau (x)) \end{array}$$ (19)

which prescribes for each disease state *x* ∈ 𝒮 both a treatment/action *a*(*x*) ∈ 𝒜 and an *examination lag time* *τ*(*x*) > 0 that denotes the time until the next diagnostic. Each determination of the patient’s virologic status incurs a *diagnostic cost* *k*_dia_.

Within this framework, controlling the disease process proceeds as follows: Assuming the patient is in state *X*_0_ = *x* ∈ 𝒮 at the initial time *t*_0_ = 0, a treatment/action *a*(*X*_0_) ∈ 𝒜 and an examination lag time *τ*(*X*_0_) > 0 are recommended. The stochastic process proceeds unobserved until time *t*_1_ = *t*_0_+*τ*(*X*_0_) when the next diagnostics are performed, revealing disease state *X*_*t*_1__ and incurring a diagnostic cost *k*_dia_. Based on the state *X*_*t*_1__, a (possibly) new treatment/action *a*(*X*_*t*_1__) and a time lapse for next examination *τ*(*X*_*t*_1__) are recommended, etc… The resulting *examination times* (*t*_0_,*t*_1_,*t*_2_,…) depend on the stochastic dynamics of the process and the applied policy. A switch of actions can only happen at examination times *t*_*j*_, when the physician changes treatment due to the diagnosed disease status *X*_*t*_*j*__.

The performance criterion for the corresponding control problem is given by:

$$\begin{array}{c} J\left(x,u\right)={𝔼}_{x}^{u}(\sum_{j=0}^{\infty }e^{-\lambda t_{j}}(C(X_{t_{j}},a\left(X_{t_{j}}\right),\tau \left(X_{t_{j}}\right))+e^{-\lambda \tau (X_{t_{j}})}k_{\mathrm{dia}})), \end{array}$$ (20)

see [25], where ${𝔼}_{x}^{u}$ stands for the expectation value with respect to the measure determined by the initial state *x* and the control *u*. The value function for this problem is given by

$$\begin{array}{c} V\left(x\right):=\underset{u\in 𝒰}{\inf}\;J\left(x,u\right) \end{array}$$ (21)

with corresponding *Bellman Equation*:

$$\begin{array}{c} V\left(x\right)=\min_{a\in 𝒜,\tau \in [0,\infty )}(\;C\left(x,a,\tau \right)+e^{-\lambda \tau }\;(k_{\mathrm{dia}}+(V^{\mathrm{tr}}\cdot T_{a,\tau })\left(x\right))), \end{array}$$ (22)

see [25] for the proof. The *Bellman Equation* can be used in order to numerically solve this optimal control problem, which requires to find an optimal treatment and an optimal *examination lag time* for each possible disease state, see S1 Text for a detailed description of the algorithm.

#### Pro-active strategy (open-loop optimal control)

In the **pro-active strategy**, no diagnostics are taken. Instead, all possible disease trajectories are anticipated in a probabilistic sense and decisions depend on the actual *probability state* *p* ∈ Ω of a patient; –i.e. the probabilities of being in either of each possible disease states *x* ∈ *S*. Given a treatment, this *probability state* of a patient evolves in a deterministic way, see Eq (2). By omitting diagnostics, the **pro-active strategy** may have the advantage of being more easily implementable in settings where resources and infrastructure would not allow for patient-specific diagnosis and treatment.

In this context, an optimal policy prescribes an action to each possible probability measure *p* ∈ Ω on the (infection) state space *S*:

$$\begin{array}{c} u:\Omega \to 𝒜,\;p\mapsto u(p)=(a(p)) \end{array}$$

with *p*[*x*]: = ℙ(*X* = *x*).

We discretize the considered time index and allow treatment changes only for certain times $t_{j}=j\cdot \bar{\tau }$, *j* ∈ ℕ, where $\bar{\tau }$ is a fixed time lag. Within such a time interval of length $\bar{\tau }$ the action remains fixed, i.e. switching a treatment is possible only after a minimum time interval $\bar{\tau }$. We denote by $p_{j}=p(j\cdot \bar{\tau })$ the probability state at these time points and set $T_{a}:=T_{a,\bar{\tau }}$ for simplicity. The state equation is then given by

$$\begin{array}{c} p_{j+1}=T_{a}\cdot p_{j}, \end{array}$$ (23)

where *a* ∈ 𝒜 is the action applied in the j^th^ interval and *p*_0_ is a fixed initial state probability vector. The transition matrix $T_{a,\bar{\tau }}$ related to the action *a* and time lag $\bar{\tau }$ is defined in Eq (3). Unlike the **diagnostic-guided strategy** where the switching times are also the observation times, for the **pro-active strategy**, the disease process is unobserved.

For the **pro-active strategy** the performance criterion entails only *state* and *action* costs but no diagnostic costs. In analogy to (20), the performance criterion is given by

$$\begin{array}{c} J\left(p_{0},u\right)={𝔼}_{p_{0}}^{u}(\sum_{j=0}^{\infty }e^{-\lambda t_{j}}C(p_{j},u_{j},\bar{\tau })) \end{array}$$ (24)

with *u*_*j*_ = *u*(*p*_*j*_). The minimization of the performance criterion *J*(*p*_0_,*u*) for a given initial distribution *p*_0_ requires to find a control *u* of infinite length (an infinite switching signal). In order to allow for a numerical solution of the above stated equation, we assume that the process is controlled for a large, but finite time horizon $\left(0,N_{ℐ}\cdot \bar{\tau }\right]$ after which a constant control *u*_∞_ ∈ 𝒜 is applied. In the current work, we used $\bar{\tau }=2$ days and $N_{ℐ}\cdot \bar{\tau }=5000$ days for a numerical solution. Thus, for the **pro-active strategy** we seek a sequence of *N*_ℐ_+1 actions (*u*_0_,*u*_1_,…*u*_*N*_ℐ_−1_,*u*_∞_) for a given initial probability distribution *p*_0_. We denote the set of all admissible controls by 𝒰. Obviously, the size of control space is |𝒰| = |𝒜|^*N*_ℐ_+1^.

Assuming that actions can only be changed for the finite time horizon $\left[0,N_{ℐ}\cdot \bar{\tau }\right]$ and an action is maintained afterwards, we derive a *Bolza Type* of performance criterion from the general formulation in Eq (24):

$$\begin{array}{c} J\left(p_{0},u\right)={𝔼}_{p_{0}}^{u}(\sum_{j=0}^{N_{ℐ}-1}e^{-\lambda t_{j}}C(p_{j},u_{j},\bar{\tau })+e^{-\lambda t_{N_{ℐ}}}C(p_{N_{ℐ}},u_{\infty },\infty )) \end{array}$$ (25)

denoting the expected costs for the infinite time horizon, given an initial distribution *p*_0_ ∈ Ω and a control *u*. The performance criterion Eq (25) for the **pro-active strategy** contains a terminal cost and a running cost, see S2 Text. Given an initial state vector *p*_0_, a control *u* ∈ 𝒰 and fixed action *u*_∞_ after the interval *N*_ℐ_, the expression can be simplified to

$$\begin{array}{c} J\left(p_{0},u\right)=\sum_{j=0}^{N_{ℐ}-1}q_{u_{j},j}^{'}\cdot p_{j}+q_{u_{\infty }}^{'}\cdot p_{N_{ℐ}} \end{array}$$ (26)

where $q_{u_{\infty }}\in {\mathbb{R}}_{+}^{\left|S\right|}$ and $q_{u_{j},j}\in {\mathbb{R}}_{+}^{\left|S\right|}$ are the terminal and the running cost vectors respectively. Now, the optimal control problem can be defined as:

$$\begin{array}{c} \begin{array}{cc} J^{*}\left(p_{0},u^{*}\right) & =\min_{u\in 𝒰}\;\sum_{j=0}^{N_{ℐ}-1}q_{u_{j},j}^{'}\cdot p_{j}+q_{u_{\infty }}^{'}\cdot p_{N_{ℐ}}\\ \text{w.r.t}\;\;p_{j+1} & =T_{u_{j}}\cdot p_{j}\\ p_{0} & =p(0)\cdot \end{array} \end{array}$$ (27)

The *Hamiltonian function* for the j^th^ interval is given by the following equation

$$\begin{array}{c} H_{j}=\xi_{j+1}^{'}\cdot T_{u_{j}}\cdot p_{j}+q_{u_{j},j}^{'}\cdot p_{j} \end{array}$$ (28)

where *ξ* is the *adjoint vector*. The adjoint equation and transversal condition are given by

$$\begin{array}{ccc} \xi_{j}^{'} & = & \xi_{j+1}^{'}\cdot T_{u_{j}}+q_{u_{j},j}^{'}\\ \xi_{N_{ℐ}}^{'} & = & q_{u_{\infty }}^{'}\cdot \end{array}$$ (29)

The *Bellman Equation* for the discrete-case [27, 55] is given by

$$\begin{array}{ccc} V(p_{j},j) & = & \min_{a\in 𝒜}(q_{a,j}^{'}\cdot p_{j}+V\left(p_{j+1},j+1\right))\\ & = & \min_{a\in 𝒜}(e^{-\lambda \cdot t_{j}}C\left(p_{j},a,\bar{\tau }\right)+V\left(p_{j+1},j+1\right))\cdot \end{array}$$ (30)

Eq (29) allows to redefine the optimal control problem Eq (27) for any *m* ∈ {0⋯*N*_ℐ_} as shown below

$$\begin{array}{c} \begin{array}{ccc} J^{*}\left(p_{0},u^{*}\right) & =\underset{u\in 𝒰}{\text{min}}\left(\xi_{m}^{'}\cdot p_{m}+\overset{m-1}{\underset{j=0}{\sum }}q_{u_{j},j}^{'}\cdot p_{j}\right) & \\ \text{w}.\text{r}.\text{t}\;\;p_{i+1} & =T_{u_{i}}\cdot p_{i} & ;p_{0}=p\left(0\right)\\ \xi_{l}^{'} & =\xi_{l+1}^{'}\cdot T_{u_{l}}+q_{u_{l},l}^{'} & ;\xi_{N_{ℐ}}^{'}=q_{u_{\infty }}^{'} \end{array} \end{array}$$ (31)

where *i* = 0…(*m*−1) and *l* = (*N*_ℐ_−1)…*m*. This formulation shows the similarity of the optimal control problem to a two point boundary value problem for a continuous case. The boundary conditions are *p*_0_ = *p*(0) and *ξ*_*N*_ℐ__ = *q*_*u*_∞__. Note that the optimal control problem needs to be solved for all possible boundary conditions for the adjoint vectors, i.e. by iterating over all possible actions for *u*_∞_.

### Numerical Solution

Solving optimal control problems is generally computation intense and may not always be achievable. Our two optimal control scenarios require different algorithms for their solution.

For computing the optimal **diagnostic-guided strategy**, we used an adapted policy iteration algorithm, see S1 Text for details.

In order to numerically compute the optimal **pro-active strategy**, we introduce a dynamic programming technique in S2 Text, which was developed for the considered performance criterion (expected discounted costs over an infinite time horizon). It has some similarity with the algorithm introduced by Hernandez-Vargas [27], which, however, considers a different performance criterion (only terminal cost).

Both algorithms were implemented in MATLAB Version 8 and parallelized, where applicable. For the dynamic programming technique in S2 Text we used the state of art solver cplex from the IBM ILOG CPLEX [56] Optimization Studio to solve embedded linear programs.

## Results

### Optimal Treatment Strategy

The optimal **diagnostic-guided strategy** is given in S1 Table. In brief, for the considered parameters (Tables 1 and 2), it is suggested to use the first-line treatment *a*_1_ in all states, except those where the virus is resistant against treatment *a*_1_, but susceptible to *a*_2_. In the later case treatment line *a*_2_ is suggested. In line with this treatment recommendation, patient monitoring is only suggested as long as the patient is infected with drug-susceptible (“wild-type”) virus. If the patient has a *h*igh or *m*edium virus load, the next diagnostic test should be within 25 days, if the patient has a ℓow/non-detectable virus load, after 152 days.

**Fig 4:**
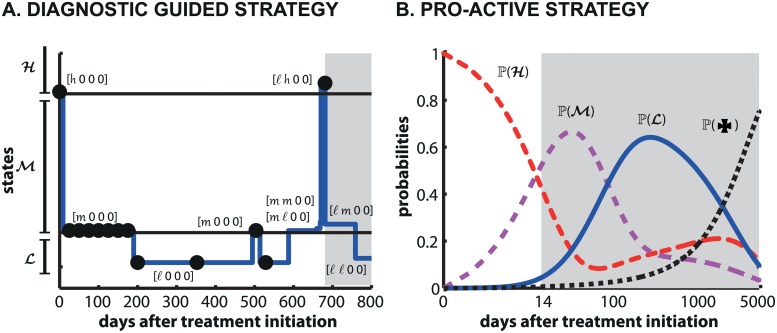
Disease progression for the diagnostic-guided strategy (individual trajectory, panel A) and pro-active strategy (probabilistic measure, panel B). The white region denotes application of treatment *a*_1_ and the gray region denotes the application of treatment *a*_2_. We assumed that the initial HIV virologic status is represented by a treatment-naive patient with high copy number of wild type virus [*n*_*C*_(WT) = *h*,*n*_*C*_(R1) = 0,*n*_*C*_(R2) = 0,*n*_*C*_(HR) = 0]. In panel **A**, the blue line represents a stochastic realization of HIV dynamics in a single individual treated with the **diagnostic-guided strategy** and black dots indicate diagnostic assessments. In the y-axis, all states belonging to the set of viral states ℋ, ℳ and 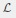 are indicated. 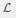 denotes an undetectable total viral load, i.e. this is the set of states for which condition *n*_*C*_(*M*) ≤ ℓ for all possible virus mutants *M* holds ([≤ℓ,≤ℓ,≤ℓ,≤ℓ]). Likewise, ℋ denotes a high total viral load, i.e. refers to all states for which for at least one viral strain *M*, *n*_*C*_(*M*) > *m*. The remaining viral states belong to ℳ. Only the initial part of the trajectory is presented (day 0–800 after treatment initiation) and details of transitions to each state are labeled for clarity. In panel **B**, the black, red, magenta and blue lines represent the probabilities of states ✠ (patient death), ℋ, ℳ and 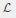 after application of the **pro-active strategy**. Note, that for the **pro-active strategy**, the x-axis is logarithmically scaled.

These results may indicate that the cost for diagnostics is too high in relation to the economic benefit resulting from more close monitoring and informed treatment adaptation (this will be discussed later in the *Discussion*). An exemplary trajectory that highlights the treatment strategy is shown in Fig 4A. The blue line indicates a *patient-specific* trajectory. The filled black marks indicate the times when a diagnostic test is performed and the background shading indicates the applied treatment (white: *a*_1_, gray shading: *a*_2_). In the example, the patient initially has a high copy number *h* of wild type (WT) virus, while none of the drug resistant viruses are present. This state is represented by the vector notation $X_{t_{0}}=[h,\;0,\;0,\;0]$. For this state, the optimal treatment policy (see S1 Table) suggests to use treatment *a*_1_ and to perform the next diagnostic test in 25 days (the second black marking in panel Fig 4A). At the next diagnostic test, the patient is in state [m,0,0,0] for which continuation of treatment *a*_1_ is recommended and the next diagnostic test is scheduled after 25 days (the 3^rd^–9^th^ black marking in panel Fig 4A). In the following, the virus remains suppressed, with a small detected ‘blip’ after about 500 days. After about 600 days of treatment, during the time lapse between diagnostic tests, the *a*_1_ resistant strain R1 emerges. Notice transitions from the state [m,0,0,0]→[m,ℓ,0,0]→[m,m,0,0], then [ℓ,m,0,0] and finally [ℓ,h,0,0] in the Fig 4A, where the copy number of *a*_1_ resistant strain R1 increases from a ℓow copy number to a *h*igh copy number (virus rebound after resistance development). At the time point of the next diagnostic (at around 700 days), the emergence of resistance is identified [ℓ,h,0,0] and a switch to treatment *a*_2_ is suggested (marked by gray region in Fig 4A). After the initiation of treatment *a*_2_, a transition to state [ℓ,ℓ,0,0] can be observed in the trajectory, which implies a decrease in the *a*_1_ resistant strain (viral suppression).

The optimal **pro-active strategy** depends on the initial *probability state* of the patient *p*_0_. We assumed that the patient is treatment naive and has *h*igh virus copy numbers, i.e. $p[h,\;0,\;0,\;0](t_{0})=1$ and $p[x](t_{0})=0$ for x∈𝒮\[h,0,0,0]. For this scenario, it is suggested to start with treatment line *a*_1_ and to switch to *a*_2_ after 14 days, which is then maintained. The trajectories of the patient *probability states* are depicted in Fig 4B. For the ease of interpretation, we illustrate only the sets of viral states 𝓛, ℳ, ℋ and patient death ✠. 𝓛 denotes an undetectable total viral load. Translated to our model, this is the set of states for which condition *n*_*C*_(*M*) ≤ ℓ for all possible virus mutants *M* holds, i.e. the current state has to fulfill [≤ℓ,≤ℓ,≤ℓ,≤ℓ] to belong to this set. Likewise ℋ denotes a high total viral load, i.e. refers to all states for which for at least one viral strain *M*, *n*_*C*_(*M*) > *m* is fulfilled. The remaining viral states belong to ℳ. One can nicely see that after approximately 260 days, maximum viral suppression may be achieved in the sense that the probability to have undetectable virus load (𝓛) is maximal (64.19%), while the patient may have intermediate viral loads ℳ with 15.57% probability and high viral loads ℋ with only 14.40% probability (the probability of death is 5.84%). After this time, it becomes more likely to fail treatment, as indicated by an increase in states ℳ and ℋ relative to 𝓛. We also assessed the sensitivity of the optimal **pro-active strategy** to variations in parameter values and found it to be fairly insensitive to parameter perturbations, see S3 Text. For comparison, we also show the dynamics for the case when no treatment switches were conducted in S4 Text in relation to the optimal **pro-active strategy**.

### Cost of Strategy

In our model, the cost incurred by a treatment strategy can be divided into two types: The direct costs, which include treatment- and diagnostic costs, and indirect costs incurred by the virologic/health status of a patient (state costs). The **pro-active strategy** does not comprise diagnostic tests, whereas the protocol for the current **standard of care** (S.O.C.), as well as the protocol used in the **HPTN052** [16], which we simulate for comparison, require viral load measurements. Currently, the expensive resistance tests are not part of the protocol for the **standard of care**, nor were they used for treatment decisions in **HPTN052**. The protocol for S.O.C. recommends changing treatment, if viral load (which is measured at month 6 and then every 12 months) is detectable and confirmed in a follow up testing after 2 months. The protocol for the **HPTN052** trial recommends changing treatment, if two consecutive viral load measurements were greater than 1000 copies/mL, 16 weeks after treatment initiation. Viral load was measured at week 2, at month 1, 2, 3 after treatment initiation and then every 3 month. The cost of virologic testing is roughly 30 US$ per test [57, 58]. In contrast to S.O.C. and **HPTN052**, the **diagnostic-guided strategy** requires drug resistance testing. We set the cost of the diagnostics for the **diagnostic-guided strategy** to 200 US$ per test, in line with the recent literature [57, 59].

**Table 3:** Expected discounted costs for an infinite time horizon.

Type of cost	Standard of care protocol [US$]	HPTN052 protocol [US$]	Pro-active strategy [US$]	Diagnostic-guided strategy [US$]
Treatment costs	1,725	1,974	2,772	1,307
Diagnostic costs	146	416	–	4,232
Total handling cost	1,871	2,390	2,772	5,539
State costs	83,770	82,210	81,047	78,319
Total cost	85,641	84,600	83,819	83,858

For each treatment strategy, the total expected discounted cost for an infinite time horizon are shown. Further, the total cost is splitted into direct cost (treatment cost and diagnostic cost) and indirect cost (state costs).

Table 3 displays the expected discounted costs for an infinite time horizon for different strategies and highlights the direct- and indirect costs of each strategy, respectively. This comparison shows that the **pro-active strategy** performs best (83,819 US$), followed closely by the **diagnostic-guided strategy** (83,858 US$), the **HPTN052 protocol** (84,600 US$) and then by the **standard of care** (85,641 US$). The total expected discounted costs for the **pro-active**- and the **diagnostic-guided strategy** are 2% less than that of the **standard of care**. The state costs (indirect cost related to patient-well being) are the major determinant of the total cost, making up roughly 98%, 97%, 97% and 93% of total cost for the S.O.C., the **HPTN052 protocol**, the **pro-active**—and the **diagnostic-guided strategy** respectively. In terms of state costs, the **diagnostic-guided strategy** performs best.

The direct costs (treatment and diagnostic costs) are highest for the **diagnostic-guided strategy** (5,539 US$) followed by the **pro-active strategy** (2,772 US$), the **HPTN052 protocol** (2,390 US$) and the **standard of care** (1,871 US$). The direct costs make up only 2%, 3%, 3% and 7% of the total costs for S.O.C., the **HPTN052 protocol**, the **pro-active** and the **diagnostic-guided strategy** respectively. The direct costs of the **pro-active** and the **diagnostic-guided strategy** are roughly 48% and 196% more than that of S.O.C.

### Patient Survival

Clearly, the primary goal of any treatment strategy is to improve and prolong the life expectancy of the treated individual. We therefore compare the distinct treatment strategies in terms of patient survival. For that purpose, we define the following term:

$$\begin{array}{c} \mathbb{P}(X_{s}=✠|\mathrm{stg}) \end{array}$$

which denotes the probability of death ✠ at time *s* given that the patient was treated according to treatment strategy stg. Given two distinct strategies; stg and a reference treatment strategy stg_ref_, the term $T_{0\to t}^{+}(\text{stg},{\text{stg}}_{\text{ref}})$ refers to the expected years of life gained (life prolongation) when the treatment strategy stg is used, relative to the reference treatment stg_ref_ at time *t* after initiation of treatment:

$$\begin{array}{c} T_{0\to t}^{+}\left(\mathrm{stg},{\mathrm{stg}}_{\mathrm{ref}}\right)=\int_{s=0}^{t}\mathbb{P}(X_{s}=✠|{\mathrm{stg}}_{\mathrm{ref}})-\mathbb{P}(X_{s}=✠|\mathrm{stg})\;ds\cdot \end{array}$$ (32)

In other words, given a patient is treated with stg and another patient is treated with stg_ref_ for time *t*, the terms $T_{0\to t}^{+}(\text{stg},{\text{stg}}_{\text{ref}})$ refers to the expected time that a patient treated with stg will live longer than the patient treated with stg_ref_.

**Fig 5:**
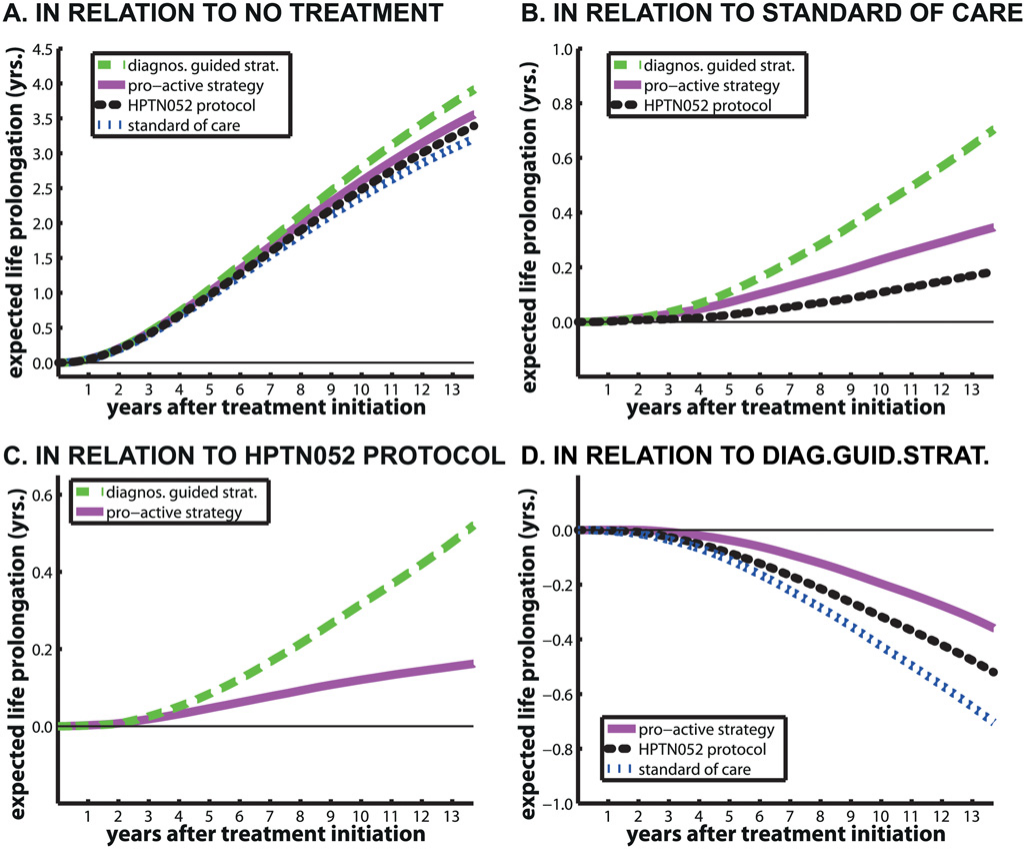
Relative expected life prolongation [years] for different treatment strategies. The purple solid lines, green dashed lines, blue dash-dotted lines and black dots represent the **pro-active strategy**-, the **diagnostic-guided strategy**, the current **standard of care** and the **HPTN052 protocol** respectively. The thin black line denotes the line of unity (no improvement/worsening). Panels A-D show the expected life-time prolongation for the distinct treatment strategies in relation to the **no treatment**, **standard of care**, the **HPTN052 protocol** and the optimal **diagnostic-guided strategy** respectively.

We compared all strategies with the following reference strategies stg_ref_: i) no medical intervention, ii) the **standard of care** treatment, iii) treatment according to the **HPTN052 protocol** and iv) the **diagnostic-guided strategy**. Fig 5A and 5D show the trajectories of expected life prolongation by different strategies in relation to i)-iv). Table 4 displays the expected life-years gained after 1 -, 2 -, 5 -, 8 -, 12—and 13.7 years of treatment respectively, where we additionally show the expected life prolongation in relation to the uninfected state.

**Table 4:** Expected relative life-time gained using different strategies.

		Expected life prolongation [years] after					
Ref. Strategy	Test Strategy	1 yr	2 yrs	5 yrs	8 yrs	12 yrs	13.7 yrs
No disease	Diag-guided strategy	-0.020	-0.070	-0.360	-0.870	-1.830	-2.300
No disease	Pro-active strategy	-0.020	-0.070	-0.400	-0.990	-2.110	-2.660
No disease	HPTN052 protocol	-0.030	-0.080	-0.450	-1.080	-2.250	-2.820
No disease	Standard of care	-0.030	-0.090	-0.470	-1.150	-2.400	-3.010
No disease	No treatment	-0.070	-0.280	-1.420	-2.980	-5.260	-6.220
No treatment	Diag-guided strategy	0.051	0.206	1.058	2.115	3.427	3.912
No treatment	Pro-active strategy	0.051	0.206	1.020	1.993	3.150	3.554
No treatment	HPTN052 protocol	0.050	0.200	0.970	1.900	3.000	3.390
No treatment	Standard of care	0.050	0.190	0.940	1.830	2.850	3.210
Standard of care	Diag-guided strategy	0.003	0.014	0.110	0.284	0.569	0.704
Standard of care	Pro-active strategy	0.004	0.014	0.072	0.163	0.292	0.345
Standard of care	HPTN052 protocol	0.002	0.007	0.026	0.070	0.149	0.184
HPTN052 protocol	Diag-guided strategy	0.002	0.007	0.084	0.214	0.420	0.520
HPTN052 protocol	Pro-active strategy	0.002	0.007	0.047	0.092	0.144	0.162

The table shows the relative expected gain in life-time for the distinct treatment strategies in comparison to a reference strategy. The reference strategies are i) no infection ii) **no treatment** iii) **standard of care** and iv) the **HPTN052 protocol**. Values were computed using Eq (32).

The first five rows of Table 4 show the expected loss-of-life-time of an HIV infected person treated with distinct strategies in relation to an HIV uninfected person. After 13.7 years, an HIV patient receiving **no treatment** lives on average 6.2 years less than a healthy person. An HIV patient receiving treatment according to S.O.C., the **pro-active strategy**, the **diagnostic-guided strategy** or according to the **HPTN052 protocol** lives on average 3, 2.66, 2.3 and 2.82 years less than a healthy person. Fig 5A shows that all treatment strategies are better than receiving **no treatment** at all and prolong the life of an HIV patient by at least 3.2 years in relation. Fig 5B shows that the **diagnostic-guided**, **pro-active strategy** and the **HPTN052 protocol** are better at increasing patient survival than the **standard of care**. Further, Fig 5C shows that the *optimal* strategies are slightly better than the **HPTN052 protocol** and Fig 5D shows that the **pro-active strategy** and the **HPTN052 protocol** are slightly worse than the **diagnostic-guided strategy**. Table 4 shows that during the initial 2–3 years of treatment, there is almost no difference between the **diagnostic-guided** and the **pro-active strategy** with regard to patient survival. After 13.7 years of treatment, the difference between the two *optimal* strategies is less than 5 month (0.358 years).

### Expected Reduction in Secondary Cases

Besides the primary goal of improving the life of the HIV patient, ‘treatment for prevention’ has gained interest in recent years. ‘Treatment for prevention’ strategies reduce onward transmission of the virus by reducing the infectiousness of HIV positive individuals. In order to measure the efficacy of the treatment strategies in preventing HIV-1 transmission, we estimated the incidence rate per 100 person-years associated with each HIV lumped state (ℓ, *m*, *h*) from a meta-analysis by Attia et al [14] (see S5 Text). The meta-analysis summarizes the outcome of 11 clinical studies on HIV-1 transmission in heterosexual sero-discordant couples, primarily from Africa.

For a strategy stg applied for a time *t*, the following equation gives a measure of the expected number of secondary cases/transmissions per survivor

$$\begin{array}{c} {𝔼}_{0\to t}\left(\mathrm{transm}\cdot |\mathrm{stg}\wedge \neg ✠\right)=\int_{s=0}^{t}\frac{\sum_{x}\mathbb{P}\left(X_{s}=x|\mathrm{stg}\right)\cdot 𝕀\mathbb{R}\left(x\right)}{1-\mathbb{P}(X_{s}=✠)}ds \end{array}$$ (33)

where 𝕀ℝ(*x*) is the incidence rate per 100 person-years for a state *x* in our virus dynamics model, as explained in S5 Text and given in Table 1. Given two strategies, stg_1_ and stg_ref_, the percentage of potential infections prevented by strategy stg_1_ in comparison to the reference strategy stg_ref_ is given by the quotient:

$$\begin{array}{c} \%\text{transmissions prevented until}t=100\cdot (1-\frac{{𝔼}_{0\to t}\left(\mathrm{transm}\cdot |{\mathrm{stg}}_{1}\wedge \neg ✠\right)}{{𝔼}_{0\to t}\left(\mathrm{transm}\cdot |{\mathrm{stg}}_{\mathrm{ref}}\wedge \neg ✠\right)}) \end{array}$$ (34)

**Fig 6:**
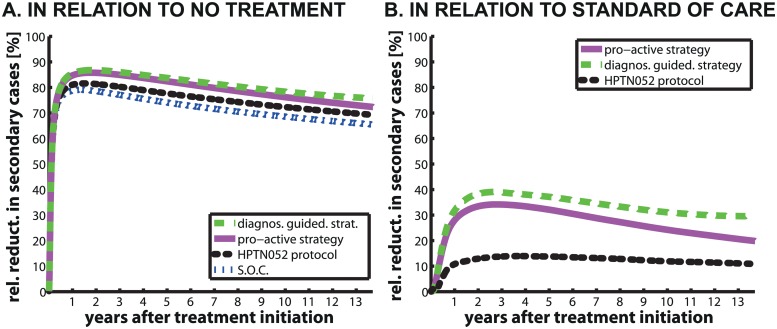
Comparison of the relative reduction of secondary cases per survivor. The purple solid, green dashed, blue dash-dotted lines and black dots represent the expected relative reduction of secondary cases per survivor by the **pro-active-**, the **diagnostic-guided strategy**, S.O.C. and in the **HPTN052 protocol**. In panel A, the reference strategy is **no treatment** and in panel B it is S.O.C.

We computed the expected reduction of secondary cases for different strategies taking either **no treatment** or the current **standard of care** as the reference strategy. In comparison to **no treatment**, the maximal reduction of secondary cases for the **pro-active** -, the **diagnostic-guided strategy**, the **HPTN052 protocol** and S.O.C. are achieved roughly 1.5–3 years after treatment initiation with values of 86%, 87%, 82% and 79% respectively, see Fig 6A. The relative reduction of secondary cases per survivor for the **diagnostic-guided** and the **pro-active strategy** are very similar, with an increase for the first 2 years, followed by a slow decline (see Fig 6A and Table 5). The relative reduction of secondary cases per survivor for the **HPTN052 protocol** is slightly better than that of S.O.C, with a tendency to decline over time, see Table 5. Note, that the computed relative reduction of secondary cases with the **HPTN052 protocol** was 82% (Table 5), which is slightly lower than the reported relative reduction of transmission events in the actual HPTN052 study [16] (reduction of 96% of linked and 89% of total transmission events). We have discussed reasons for this apparent under-prediction later in the manuscript. The difference between the optimal strategies (**diagnostic-guided** and the **pro-active strategy**) and S.O.C. becomes evident, when looking at the relative risk reduction by the optimal treatment strategies in relation to S.O.C. in Fig 6B. The reduction in secondary cases per survivor by the optimal strategies in comparison to S.O.C. is highest at the beginning and then slowly decreases over time.

**Table 5:** Expected relative reduction of secondary cases per survivor using different treatment strategies after different treatment durations.

		relative reduction in secondary cases [%]					
Ref. strategy	Test strategy	1 yr	2 yrs	3.5 yrs	5 yrs	8 yrs	13.7 yrs
No treatment	Diagnostic-guided strategy	85.52	86.76	85.42	83.71	80.33	75.72
No treatment	Pro-active strategy	84.72	85.72	84.34	82.41	78.54	72.35
No treatment	HPTN052 protocol	81.01	81.32	79.61	77.66	74.29	69.25
No treatment	Standard of care	78.77	78.55	76.31	74.08	70.50	65.50
Standard of care	Diagnostic-guided strategy	31.79	38.29	37.45	37.15	33.31	29.61
Standard of care	Pro-active strategy	28.04	33.45	33.88	32.11	27.25	19.85
Standard of care	HPTN052 protocol	10.83	12.94	13.93	13.79	12.83	10.85

The table shows the expected relative reduction in secondary cases for different strategies in comparison to **no treatment** and S.O.C. after different treatment durations. Values were computed using Eq (34).

## Discussion

The main aim of this work was to develop a rigorous mathematical framework that allows to compare different treatment paradigms in terms of monetary costs, treatment benefit and efficacy for ‘treatment for prevention’. It was previously stated [60], that the durability of ‘treatment for prevention’ should be assessed. Our simulations over a long time horizon (up to 5000 days/13.7 years) indicate that the effect of ‘treatment for prevention’ is significant and remains relatively stable beyond the time horizon typically assessed in clinical studies, see Fig 6A and Table 5, and that it may even be improved. We estimated that a **standard of care** therapy in e.g. South Africa can achieve a 66–79% reduction of HIV-1 onward transmission, in comparison to delivering **no treatment**. We also implemented the **HPTN052 protocol**, as stated in [16] and predicted that it would achieve up to 82% reduction of HIV-1 transmission, being more effective than the current **standard of care**, as shown in Fig 6B.

Statistical assessment of the actual HPTN052 trial [16] yielded estimates for the relative reduction of transmission of 96% for linked transmission and 89% for any transmission. Our simulated **HPTN052 protocol** yielded a 82% reduction of onwards transmission, which is within the confidence range of the reported estimates (CI: 73–99% for linked transmission and CI: 68–96% for any transmission) [16]. Note, that only one linked transmission event (1/1585 person-years) was observed in the early therapy arm of HPTN052 [16], giving rise to the statistical uncertainty in the reported estimate. Nevertheless, our simulations may under-predict the efficacy of HPTN052 due to several factors:

- The reported treatment efficacy in the HPTN052 study was higher than predicted by our model: Virologic failure was only observed in 5% of participants in the early-therapy group of HPTN052, possibly explaining the difference between the outcome of the simulation vs. the clinical trial.
- Despite only 5% failing to suppress the virus in the HPTN052 study, 66% initiated a second line therapy [16], meaning that a significant proportion of patients switched treatment *before/without* virologic failure. In our simulations of the **HPTN052 protocol**, patients only switched treatment when they showed signs of virologic failure. However, one may speculate that these treatment switches *before/without* virologic failure may have an impact on the long-term viral suppression that could be similar to a pro-active treatment switch.
- The primary measurable endpoint of the HPTN052 study was the infection of the sero-discordant partner. Onward transmissions to other individuals could not be quantified for obvious reasons.

While a number of trials are now underway to confirm the results of HPTN052, see e.g. [61, 62], our *in silico* approach specifically addresses the need for an improved treatment strategy, particularly taking affordability into account, which suggests strategies that are suitable for scaling up.

Our work may indicate that if ‘treatment for prevention’ is scaled up and implemented using the current **standard of care** treatment strategy, its efficacy may not be as high as expected from HPTN052. Unlike in HPTN052, where monitoring of treatment success (viral suppression) and timely execution of treatment changes were realized, in resource-constrained countries close patient monitoring is currently not implemented in a routine setting and may be difficult to realize due to infrastructural and economic requirements.

Two alternative strategies for the immediate initiation of therapy were assessed in our work that take into account the mentioned limitations. Both suggested strategies (the **diagnostic-guided strategy** and the **pro-active strategy**) yielded better results in our simulations in terms of the reduction of onward transmission (see Table 5) at a lower price (Table 3). Both *optimal* strategies could yield a 72–87% reduction in HIV onward transmission in comparison to **no treatment**, see Fig 6A and Table 5. In comparison to the **standard of care**, we estimated that the **diagnostic-guided strategy** and the **pro-active strategy** could yield another 33–38% reduction of onward transmission after 2 years of treatment, but the advantages of the **diagnostic-guided strategy** and the **pro-active strategy** over the **standard of care** slowly declined over time, see Fig 6B. This indicates that both *optimal* strategies have a particular strength in reducing early transmissions (shortly after treatment initiation) in comparison to the current **standard of care**. This may be of particular utility, if transmission occurred primarily during early infection [63, 64]. In our work, we did not take behavioral factors into account, which would lead to a time-dependency of the infection rate. Rather, we assumed that the infection rate 𝕀ℝ(*x*) is constant over time, but dependent on the total virus load as reported earlier [14, 15, 19–21]. If transmission would primarily take place during an early infection, the advantages of the **diagnostic-guided strategy** and the **pro-active strategy** over the **standard of care** would be even more pronounced than indicated in Fig 6B.

The *optimal* **diagnostic-guided strategy** suggested *patient-specific* diagnostics, i.e. dependent on the patient’s virologic status (see S1 Table), unlike fixed intervals as in S.O.C, or the protocol stated in [16]. In summary, the *optimal* **diagnostic-guided strategy** suggests to take frequent diagnostics (≈ every month) if the patient is infected with a *h*igh or *m*edium number of treatment-susceptible virus and less frequent (≈ every 5 month) diagnostics if the patient is infected with a ℓow/undetectable number of virus. No diagnostics are recommended for the remaining virologic states. Altogether, a very sparse diagnostic schedule for individual patients is suggested. Previous work [25] indicated that price reductions for the diagnostic tests would yield a better patient-outcome, which indicates that available drugs may not be utilized optimally in resource-poor settings, because diagnostics are currently too expensive. Of note is the fact that albeit treatment being available at very low expense (due to negotiations by the Clinton Foundation [65]), diagnostics may not be.

Furthermore, we suspected that allowing treatment change only *after* diagnostic confirmation of treatment failure (i.e. some time after drug resistance has occurred) may limit future treatment options [34]. Since the *optimal* **diagnostic-guided strategy** suggested rare diagnostics, and because it only allows to change treatment *after* resistance is detectable, we evaluated **pro-active switching strategies**. Note, that pro-active treatment switching strategies tested in the clinic increased virologic suppression and lowered rates of drug resistance emergence in HIV-1, when compared to conventional strategies [66, 67]. Similar strategies are also used against bacterial infections and cancers.

**Fig 7:**
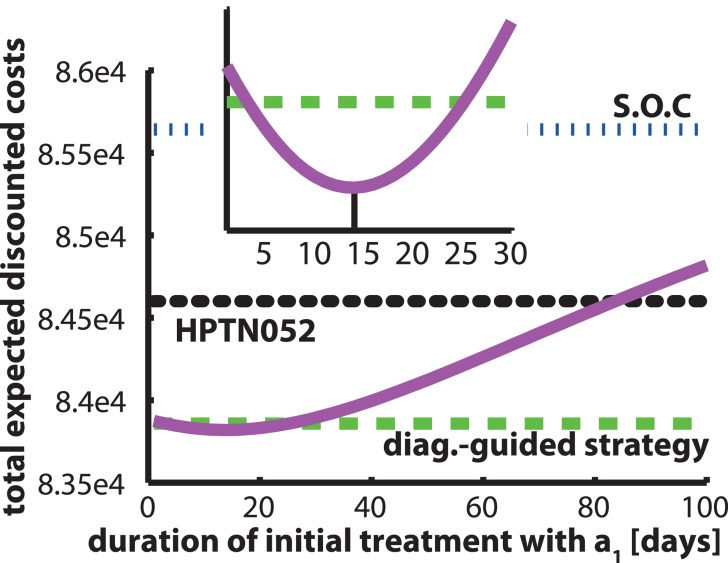
Sensitivity of the pro-active strategy with respect to timing of the treatment switch. The purple solid line represents the total expected discounted costs for the **pro-active strategy** with respect to the switching time (shown on the x-axis). The horizontal green dashed, blue dash-dotted lines and black dots represent the total expected discounted costs for the optimal **diagnostic-guided strategy**, S.O.C. and in the **HPTN052 protocol**. The inset shows a zoom into the first 30 days after treatment initiation.

The computed **pro-active strategy** suggests a single treatment change *without* prior ascertainment of the viral status within a treated patient. Surprisingly, this strategy could yield comparable outcomes in terms of monetary costs, patient health and reduction of onward transmission, see Tables 3–5 and Figs 5 and 6. Our work thus indicates that **pro-active strategies**, may be as effective as diagnostically-driven ones, when diagnostics are expensive or inaccessible. Note, that unlike other *optimal* control approaches, i.e. [28] that suggest infinitely fast switching between regimens to mitigate drug resistance emergence, our predicted **pro-active strategy** actually only recommends a single treatment change, which is clinically more realistic. We also analyzed the sensitivity of the **pro-active strategies** with respect to the timing of this switch (see Fig 7). The graphic illustrates, that the switch is optimal after 14 days, however the difference in the performance measure is marginal, as long as the treatment switch is performed before ≈ 30 days (1 month) after treatment initiation.

Obviously, pragmatic and clinical considerations need to be taken into account to translate our results into practice. Also, several assumptions have been made, which require careful evaluation. For example, we used a very coarse-grained model of HIV within-host dynamics, which was required to enable the numerical computation of optimal controls, particularly for the closed-loop system employed in the **diagnostic-guided strategy**. Most models of viral dynamics, e.g. [33, 68, 69], were developed to accurately predict short-term viral dynamics after drug application and are unable to predict virologic failure after long time intervals, in contrast to our coarse-grained model, which was developed and parameterized in order to predict short-term viral dynamics as well as virologic failure after very long time-intervals, see e.g. Figs 2 and 3. It is therefore more suitable than existing models in estimating the long-term response to antiviral treatment. However, in the future we will focus on developing more elaborated HIV-models and on algorithms to solve the control problem for the chemical master equation directly, without state-space lumping. Note, that other computationally efficient numerical approaches, such as model predictive control [30], could be used to approximate the optimal treatment strategies. However, there is no guarantee that the computed control using these approaches will be *optimal*.

In our approach, we modeled treatment change as a switched system, which neglects the pharmacokinetics of the distinct drugs [10, 70–72] and may only indirectly reflect drug adherence in an average population (drug efficacy *η* is a constant term in our model). Neglecting pharmacokinetics may, however, be a justifiable step in this modeling exercise, because of the considered time-scales (on the order of years), and also because optimizing e.g. drug adherence was not an objective of this study. However, if the main interest is for example in optimizing the switch between two treatment lines by optimal dosing in order to prevent time frames of insufficient viral suppression or drug over-exposure, or to include *patient-specific* or time-dependent drug adherence, we advise to consider a different control system, for example [73]. Within such a framework, monitoring (e.g. viral load assessment) may also be incorporated as a tool to assess individual drug adherence and to allocate resources to improve it.

We did not explicitly consider costs related to contraindications caused by the treatment. For example, the second treatment line *a*_2_ may be less tolerable. Mathematically, this can be modeled in terms of increased treatment costs for *a*_2_, in comparison to the first treatment line *a*_1_. In order to test the sensitivity of the optimal **pro-active strategy** to this parameter, we conducted the necessary computations in S3 Text and found that the computed strategy was fairly insensitive to changes in treatment costs. This may indicate that the benefits of the treatment switch outweigh these potential shortcomings.

Also, we did not include screening costs or the costs of the initial virologic assessment, thus our calculations refer to the public health costs that accrue *from the start* of HIV treatment. These costs will, however, only enter as a constant to each of the tested strategies and will not change the results beyond the addition of this constant to the values stated in Table 3. Additional costs (personnel, infrastructure, transportation) may come along with diagnostic assessments. It is likely that hidden costs for diagnostics are substantial. With a higher cost of diagnosis, the **pro-active strategy** may outperform the **diagnostic-guided strategy**, which may suggest an even less frequent diagnostic schedule, supporting our claim that current diagnostics may be too expensive to be appropriately used.

We used the price of a drug resistance test (*k*_dia_ ≈ 200 US$ [57, 59]) to account for diagnostics in the **diagnostic-guided strategy**. This had the following reason: Current guidelines recommend to measure the total virus load [13] and to switch treatment, if, based on this partial information, virologic failure is anticipated. As reported by others [57], this may lead to unnecessary treatment switches. In contrast, a resistance test directly informs the physician about the necessity of treatment change. Mathematically, partial information, i.e. the *total virus load*, would lead to a distinct control framework, namely Partially Observable Markov Decision Processes (POMDP) [74], which are extremely challenging to solve, particularly for larger models like the one used herein (Fig 1). In POMDPs, partial information may be mapped into a ‘*believed*’ full virologic status, for example *observing* a *h*igh total virus load *may* be due to some resistance development, e.g. the viral state [ℓ,h,0,0] with some probability. However, it is hard for us formalize the physicians intuition (i.e. the relation between observation, belief and truth) regarding this ‘mapping’ of partial measurements to viral states *x*.

As a primary outcome of our modeling exercise, we estimated the expected *relative* number of secondary infections prevented (Table 5 and Fig 6); -unlike many other approaches (summarized in [18]), which take the *absolute* number of secondary cases into account. Estimating *absolute* numbers of secondary cases would require to model complex behaviors, i.e. sexual relationships, etc. over time, for which we do not have data for validation, nor was it the main focus of the current work. This also prevents us from predicting the course of the epidemic or deriving its reproductive number *R*_0_ in relation to distinct treatment strategies. However, the primary aim of this study was to compare the efficacy of different treatment strategies, which is nicely quantified in terms of the expected *relative* number of secondary infections prevented. Note, that this relative estimate requires no assumptions on the underlying transmission dynamics, except that it assumes that these dynamics are similar for a tested strategy versus its comparator.

In addition to insights in HIV ‘treatment for prevention’ strategies, the developed mathematical/control theoretic framework may already be applicable to many medical phenomena. Further developments may improve its applicability to even more complex processes, which can be accurately described by intrinsically stochastic dynamics. For example, the open-loop optimal control approach (used to determine the optimal **pro-active** strategy) may be turned into a closed-loop system, if diagnostics are taken from time-to-time to determine the viral state of a patient, i.e. *p*[*x*](*t*_*j*_). Also, the closed-loop system that requires state determination (the **diagnostic-guided strategy**) may be combined with the open-loop system in order to allow for pro-active treatment changes in between diagnostic assessments.

## Supporting Information

S1 Text The supplementary text contains details of the algorithm for solving the closed-loop control system, as well as the pseudo-code. (PDF)

S2 Text The supplementary text contains details on the algorithm for solving the open-loop control system, as well as the pseudo-code. (PDF)

S3 Text The supplementary text contains an analysis of the sensitivity of the optimal **pro-active strategy** with respect to parameter variations. (PDF)

S4 Text The supplementary text contains the viral dynamics for constant treatment (no switches) in relation to the optimal **pro-active strategy**. (PDF)

S5 TextThe supplementary text contains details on the calculation of the incidence rate 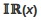 from the lumped states of our HIV-dynamics model, including a comparison with experimental data.(PDF)

S1 Table The supplementary table contains the optimal policy for the **diagnostic-guided strategy**. The first entry corresponds to the dead patient ✠.
(TXT)

## References

[1] Global report: UNAIDS report on the global AIDS epidemic 2013 (available at http://www.unaids.org/en/media/unaids/contentassets/documents/epidemiology/2013/gr2013/ accessed on 15-may-2014).

[2] Buzon MJ, Sun H, Li C, Shaw A, Seiss K, et al (2014) HIV-1 persistence in CD4+ T cells with stem cell-like properties. Nat Med 20: 139–142. 10.1038/nm.3445 24412925 PMC3959167

[3] Blankson JN, Persaud D, Siliciano RF (2002) The challenge of viral reservoirs in HIV-1 infection. Annu Rev Med 53: 557–593. 10.1146/annurev.med.53.082901.104024 11818490

[4] Fletcher CV, Staskus K, Wietgrefe SW, Rothenberger M, Reilly C, et al (2014) Persistent HIV-1 replication is associated with lower antiretroviral drug concentrations in lymphatic tissues. Proc Natl Acad Sci USA 111: 2307–2312. 10.1073/pnas.1318249111 24469825 PMC3926074

[5] von Kleist M, Metzner P, Marquet R, Schütte C (2012) HIV-1 polymerase inhibition by nucleoside analogs: cellular- and kinetic parameters of efficacy, susceptibility and resistance selection. PLoS Comput Biol 8: e1002359 10.1371/journal.pcbi.1002359 22275860 PMC3261923

[6] Grant RM, Lama JR, Anderson PL, McMahan V, Liu AY, et al (2010) Preexposure chemoprophylaxis for HIV prevention in men who have sex with men. N Engl J Med 363: 2587–2599. 10.1056/NEJMoa1011205 21091279 PMC3079639

[7] Thigpen MC, Kebaabetswe PM, Paxton LA, Smith DK, Rose CE, et al (2012) Antiretroviral preexposure prophylaxis for heterosexual HIV transmission in Botswana. N Engl J Med 367: 423–434. 10.1056/NEJMoa1110711 22784038

[8] Baeten JM, Donnell D, Ndase P, Mugo NR, Campbell JD, et al (2012) Antiretroviral prophylaxis for HIV prevention in heterosexual men and women. N Engl J Med 367: 399–410. 10.1056/NEJMoa1108524 22784037 PMC3770474

[9] Choopanya K, Martin M, Suntharasamai P, Sangkum U, Mock PA, et al (2013) Antiretroviral prophylaxis for HIV infection in injecting drug users in Bangkok, Thailand (the Bangkok Tenofovir Study): a randomised, double-blind, placebo-controlled phase 3 trial. Lancet 381: 2083–2090. 10.1016/S0140-6736(13)61127-7 23769234

[10] Duwal S, Schütte C, von Kleist M (2012) Pharmacokinetics and pharmacodynamics of the reverse transcriptase inhibitor tenofovir and prophylactic efficacy against HIV-1 infection. PLoS One 7: e40382 10.1371/journal.pone.0040382 22808148 PMC3394807

[11] Nichols BE, Baltussen R, van Dijk JH, Thuma PE, Nouwen JL, et al (2014) Cost-Effectiveness of PrEP in HIV/AIDS Control in Zambia: A Stochastic League Approach. J Acquir Immune Defic Syndr 66: 221–228. 24694930 10.1097/QAI.0000000000000145

[12] World Health Organization (WHO). Global update on HIV treatment 2013: Results, Impact and Opportunities (available at http://www.who.int/hiv/pub/progressreports/update2013/en/ accessed on 02-june-2014).

[13] The South African Anti Retroviral Treatment Guidelines 2013, Version 14 (available at http://www.sahivsoc.org/practise-guidelines/national-dept-of-health-guidelines accessed 15-may-2014).

[14] Attia S, Egger M, Müller M, Zwahlen M, Low N (2009) Sexual transmission of HIV according to viral load and antiretroviral therapy: systematic review and meta-analysis. AIDS 23: 1397–1404. 10.1097/QAD.0b013e32832b7dca 19381076

[15] Hughes JP, Baeten JM, Lingappa JR, Magaret AS, Wald A, et al (2012) Determinants of percoital-act HIV-1 infectivity among african HIV-1-serodiscordant couples. J Infect Dis 205: 358–365. 10.1093/infdis/jir747 22241800 PMC3256946

[16] Cohen MS, Chen YQ, McCauley M, Gamble T, Hosseinipour MC, et al (2011) Prevention of HIV-1 infection with early antiretroviral therapy. N Engl J Med 365: 493–505. 10.1056/NEJMoa1105243 21767103 PMC3200068

[17] Cohen J (2011) Breakthrough of the year. HIV treatment as prevention. Science 334: 1628. 10.1126/science.334.6063.1628 22194547

[18] Eaton JW, Johnson LF, Salomon JA, Bärnighausen T, Bendavid E, et al (2012) HIV treatment as prevention: systematic comparison of mathematical models of the potential impact of antiretroviral therapy on HIV incidence in South Africa. PLoS Med 9: e1001245 10.1371/journal.pmed.1001245 22802730 PMC3393664

[19] Fideli US, Allen SA, Musonda R, Trask S, Hahn BH, et al (2001) Virologic and immunologic determinants of heterosexual transmission of human immunodeficiency virus type 1 in Africa. AIDS Res Hum Retroviruses 17: 901–910. 10.1089/088922201750290023 11461676 PMC2748905

[20] Quinn TC, Wawer MJ, Sewankambo N, Serwadda D, Li C, et al (2000) Viral load and heterosexual transmission of human immunodeficiency virus type 1. Rakai Project Study Group. N Engl J Med 342: 921–929. 10.1056/NEJM200003303421303 10738050

[21] Lingappa JR, Hughes JP, Wang RS, Baeten JM, Celum C, et al (2010) Estimating the impact of plasma HIV-1 RNA reductions on heterosexual HIV-1 transmission risk. PLoS One 5: e12598 10.1371/journal.pone.0012598 20856886 PMC2938354

[22] Hosseinipour MC, Gupta RK, Zyl GV, Eron JJ, Nachega JB (2013) Emergence of HIV drug resistance during first- and second-line antiretroviral therapy in resource-limited settings. J Infect Dis 207 Suppl 2: S49–S56. 10.1093/infdis/jit107 23687289 PMC3708738

[23] Hecht R, Bollinger L, Stover J, McGreevey W, Muhib F, et al (2009) Critical choices in financing the response to the global HIV/AIDS pandemic. Health Aff (Millwood) 28: 1591–1605. 10.1377/hlthaff.28.6.1591 19887401

[24] Bärnighausen T, Salomon JA, Sangrujee N (2012) HIV treatment as prevention: issues in economic evaluation. PLoS Med 9: e1001263 10.1371/journal.pmed.1001263 22802743 PMC3393650

[25] Winkelmann S, Schütte C, von Kleist M (2014) Markov control processes with rare state observation: Theory and application to treatment scheduling in HIV-1. Communications in Mathematical Sciences 12: 859–77. 10.4310/CMS.2014.v12.n5.a4

[26] Luo R, Piovoso MJ, Martinez-Picado J, Zurakowski R (2011) Optimal antiviral switching to minimize resistance risk in HIV therapy. PloS one 6: e27047 10.1371/journal.pone.0027047 22073250 PMC3207836

[27] Hernandez-Vargas E, Colaneri P, Middleton R, Blanchini F (2011) Discrete-time control for switched positive systems with application to mitigating viral escape. International Journal of Robust and Nonlinear Control 21: 1093–1111. 10.1002/rnc.1628

[28] Hernandez-Vargas EA, Colaneri P, Middleton RH (2013) Optimal therapy scheduling for a simplified HIV infection model. Automatica 49: 2874–2880. 10.1016/j.automatica.2013.06.001

[29] Cardozo EF, Zurakowski R (2012) Robust closed-loop minimal sampling method for HIV therapy switching strategies. IEEE Transactions on Bio-Medical Engineering 59: 2227–2234. 10.1109/TBME.2012.2201479 22652153 PMC3467342

[30] Hernandez-Vargas EA, Colaneri P, Middleton RH (2014) Switching strategies to mitigate HIV mutation. IEEE Transactions on Control Systems Technology 22: 1623–1628. 10.1109/TCST.2013.2280920

[31] Rouzine IM, Rodrigo A, Coffin JM (2001) Transition between stochastic evolution and deterministic evolution in the presence of selection: general theory and application to virology. Microbiol Mol Biol Rev 65: 151–185. 10.1128/MMBR.65.1.151-185.2001 11238990 PMC99023

[32] Althaus CL, Boer RJD (2008) Dynamics of immune escape during HIV/SIV infection. PLoS Comput Biol 4: e1000103 10.1371/journal.pcbi.1000103 18636096 PMC2423483

[33] von Kleist M, Menz S, Huisinga W (2010) Drug-class specific impact of antivirals on the reproductive capacity of HIV. PLoS computational biology 6: e1000720 10.1371/journal.pcbi.1000720 20361047 PMC2845651

[34] von Kleist M, Menz S, Stocker H, Arasteh K, Schütte C, et al (2011) HIV quasispecies dynamics during pro-active treatment switching: Impact on multi-drug resistance and resistance archiving in latent reservoirs. PLoS One 6: e18204 10.1371/journal.pone.0018204 21455303 PMC3063788

[35] Wilkinson DJ (2006) Stochastic Modelling for Systems Biology. Chapman & Hall/CRC.

[36] Allen LJS (2011) An Introduction to Stochastic Processes with Applications to Biology. Chapman & Hall/CR.

[37] Gillespie DT (1977) Exact stochastic simulation of coupled chemical reactions. J Phys Chem 81: 2340–61. 10.1021/j100540a008

[38] Pahle J (2009) Biochemical simulations: stochastic, approximate stochastic and hybrid approaches. Brief Bioinform 10: 53–64. 10.1093/bib/bbn050 19151097 PMC2638628

[39] Menz S, Latorre J, Schütte C, Huisinga W (2012) Hybrid stochastic-deterministic solution of the chemical master equation. SIAM Multiscale Modelling and Simulation 10: 1232–62. 10.1137/110825716

[40] Hasenauer J, Wolf V, Kazeroonian A, Theis FJ (2013) Method of conditional moments (MCM) for the Chemical Master Equation: A unified framework for the method of moments and hybrid stochastic-deterministic models. J Math Biol. 23918091 10.1007/s00285-013-0711-5

[41] Markowitz M, Louie M, Hurley A, Sun E, Mascio MD, et al (2003) A novel antiviral intervention results in more accurate assessment of human immunodeficiency virus type 1 replication dynamics and T-cell decay in vivo. J Virol 77: 5037–5038. 10.1128/JVI.77.8.5037-5038.2003 12663814 PMC152136

[42] Fischer M, Joos B, Niederst B, Kaiser P, Hafner R, et al (2008) Biphasic decay kinetics suggest progressive slowing in turnover of latently HIV-1 infected cells during antiretroviral therapy. Retrovirology 5: 107 10.1186/1742-4690-5-107 19036147 PMC2630982

[43] Paci P, Carello R, Bernaschi M, D’Offizi G, Castiglione F (2009) Immune control of HIV-1 infection after therapy interruption: immediate versus deferred antiretroviral therapy. BMC Infect Dis 9: 172 10.1186/1471-2334-9-172 19840392 PMC2771028

[44] Harrigan PR, Whaley M, Montaner JS (1999) Rate of HIV-1 RNA rebound upon stopping antiretroviral therapy. AIDS 13: F59–F62. 10.1097/00002030-199905280-00001 10371167

[45] Ruiz L, Martinez-Picado J, Romeu J, Paredes R, Zayat MK, et al (2000) Structured treatment interruption in chronically HIV-1 infected patients after long-term viral suppression. AIDS 14: 397–403. 10.1097/00002030-200003100-00013 10770542

[46] Arribas JR, Pozniak AL, Gallant JE, DeJesus E, Gazzard B, et al (2008) Tenofovir disoproxil fumarate, emtricitabine, and efavirenz compared with zidovudine/lamivudine and efavirenz in treatment-naive patients: 144-week analysis. J Acquir Immune Defic Syndr 47: 74–78. 10.1097/QAI.0b013e31815acab8 17971715

[47] Cooper DA, Heera J, Goodrich J, Tawadrous M, Saag M, et al (2010) Maraviroc versus efavirenz, both in combination with zidovudine-lamivudine, for the treatment of antiretroviral-naive subjects with CCR5-tropic HIV-1 infection. J Infect Dis 201: 803–813. 10.1086/650697 20151839

[48] DeJesus E, McCarty D, Farthing CF, Shortino DD, Grinsztejn B, et al (2004) Once-daily versus twice-daily lamivudine, in combination with zidovudine and efavirenz, for the treatment of antiretroviral-naive adults with HIV infection: a randomized equivalence trial. Clin Infect Dis 39: 411–418. 10.1086/424009 15307010

[49] DeJesus E, Herrera G, Teofilo E, Gerstoft J, Buendia CB, et al (2004) Abacavir versus zidovudine combined with lamivudine and efavirenz, for the treatment of antiretroviral-naive HIV-infected adults. Clin Infect Dis 39: 1038–1046. 10.1086/424009 15472858

[50] Gulick RM, Ribaudo HJ, Shikuma CM, Lalama C, Schackman BR, et al (2006) Three- vs four-drug antiretroviral regimens for the initial treatment of HIV-1 infection: a randomized controlled trial. JAMA 296: 769–781. 10.1001/jama.296.7.769 16905783

[51] Hicks C, King MS, Gulick RM, White AC, Eron JJ, et al (2004) Long-term safety and durable antiretroviral activity of lopinavir/ritonavir in treatment-naive patients: 4 year follow-up study. AIDS 18: 775–779. 10.1097/00002030-200403260-00008 15075512

[52] Smith KY, Patel P, Fine D, Bellos N, Sloan L, et al (2009) Randomized, double-blind, placebo-matched, multicenter trial of abacavir/lamivudine or tenofovir/emtricitabine with lopinavir/ritonavir for initial HIV treatment. AIDS 23: 1547–1556. 10.1097/QAD.0b013e32832cbcc2 19542866

[53] Perelson AS, Essunger P, Cao Y, Vesanen M, Hurley A, et al (1997) Decay characteristics of HIV-1-infected compartments during combination therapy. Nature 387: 188–191. 10.1038/387188a0 9144290

[54] Kates J, Boortz K, Lief E, Avila C, Gobet B (2012) Financing the Response to AIDS in Lowand Middle- Income Countries: International Assistance from the G8, European Commission and Other Donor Governments in 2009. Technical report, UNAIDS.

[55] Lenhart S, Workman JT (2007) Optimal control applied to biological models. CRC Press.

[56] IBM ILOG CPLEX (available at http://www-01.ibm.com/software/ accessed 15-may-2014).

[57] Rosen S, Long L, Sanne I, Stevens WS, Fox MP (2011) The net cost of incorporating resistance testing into HIV/AIDS treatment in South Africa: a Markov model with primary data. J Int AIDS Soc 14: 24 10.1186/1758-2652-14-24 21575155 PMC3119176

[58] Steegen K, Luchters S, Cabooter ND, Reynaerts J, Mandaliya K, et al (2007) Evaluation of two commercially available alternatives for HIV-1 viral load testing in resource-limited settings. J Virol Methods 146: 178–187. 10.1016/j.jviromet.2007.06.019 17686534

[59] Elliott JH, Lynen L, Calmy A, Luca AD, Shafer RW, et al (2008) Rational use of antiretroviral therapy in low-income and middle-income countries: optimizing regimen sequencing and switching. AIDS 22: 2053–2067. 10.1097/QAD.0b013e328309520d 18753937

[60] Chen YQ, Masse B, Wang L, Ou SS, Li X, et al (2012) Statistical considerations for the HPTN 052 study to evaluate the effectiveness of early versus delayed antiretroviral strategies to prevent the sexual transmission of HIV-1 in serodiscordant couples. Contemp Clin Trials 33: 1280–1286. 10.1016/j.cct.2012.07.007 22813645 PMC3468650

[61] Hayes R, Ayles H, Beyers N, Sabapathy K, Floyd S, et al (2014) HPTN 071 (PopART): rationale and design of a cluster-randomised trial of the population impact of an HIV combination prevention intervention including universal testing and treatment - a study protocol for a cluster randomised trial. Trials 15: 57 10.1186/1745-6215-15-57 24524229 PMC3929317

[62] Iwuji CC, Orne-Gliemann J, Tanser F, Boyer S, Lessells RJ, et al (2013) Evaluation of the impact of immediate versus WHO recommendations-guided antiretroviral therapy initiation on HIV incidence: the ANRS 12249 TasP (Treatment as Prevention) trial in Hlabisa sub-district, KwaZulu-Natal, South Africa: study protocol for a cluster randomised controlled trial. Trials 14: 230 10.1186/1745-6215-14-230 23880306 PMC3750830

[63] Brenner BG, Roger M, Routy JP, Moisi D, Ntemgwa M, et al (2007) High rates of forward transmission events after acute/early HIV-1 infection. J Infect Dis 195: 951–959. 10.1086/512088 17330784

[64] Recordon-Pinson P, Anies G, Bruyand M, Neau D, Morlat P, et al (2009) HIV type-1 transmission dynamics in recent seroconverters: relationship with transmission of drug resistance and viral diversity. Antivir Ther 14: 551–556. 19578240

[65] The Clinton Health Access Initiative (2011). Antiretroviral (ARV) ceiling price list (available at http://www.clintonfoundation.org, accessed 22-sept-2014).

[66] Martinez-Picado J, Negredo E, Ruiz L, Shintani A, Fumaz CR, et al (2003) Alternation of antiretroviral drug regimens for HIV infection. A randomized, controlled trial. Ann Intern Med 139: 81–89. 10.7326/0003-4819-139-2-200307150-00007 12859157

[67] Negredo E, Paredes R, Peraire J, Pedrol E, Côté H, et al (2004) Alternation of antiretroviral drug regimens for HIV infection. efficacy, safety and tolerability at week 96 of the Swatch study. Antivir Ther 9: 889–893. 15651747

[68] Perelson AS, Nelson PW (1999) Mathematical analysis of HIV-1 dynamics in vivo. SIAM Review 41: 3–44. 10.1137/S0036144598335107

[69] Sedaghat AR, Dinoso JB, Shen L, Wilke CO, Siliciano RF (2008) Decay dynamics of HIV-1 depend on the inhibited stages of the viral life cycle. Proc Natl Acad Sci U S A 105: 4832–4837. 10.1073/pnas.0711372105 18362342 PMC2290747

[70] Frank M, von Kleist M, Kunz A, Harms G, Schütte C, et al (2011) Quantifying the impact of nevirapine-based prophylaxis strategies to prevent mother-to-child transmission of HIV-1: a combined pharmacokinetic, pharmacodynamic, and viral dynamic analysis to predict clinical outcomes. Antimicrob Agents Chemother 55: 5529–5540. 10.1128/AAC.00741-11 21947390 PMC3232796

[71] von Kleist M, Huisinga W (2009) Pharmacokinetic-pharmacodynamic relationship of NRTIs and its connection to viral escape: an example based on zidovudine. Eur J Pharm Sci 36: 532–543. 10.1016/j.ejps.2008.12.010 19150497

[72] Dixit NM, Perelson AS (2004) Complex patterns of viral load decay under antiretroviral therapy: influence of pharmacokinetics and intracellular delay. J Theor Biol 226: 95–109. 10.1016/j.jtbi.2003.09.002 14637059

[73] Imran M, Smith HL (2014) A model of optimal dosing of antibiotic treatment in biofilm. Math Biosci Eng 11: 547–571. 10.3934/mbe.2014.11.547 24506551

[74] Bäuerle N, Rieder U (2011) Markov Decision Processes with Applications to Finance, Springer, chapter Partially Observable Markov Decision Processes. pp. 147–174.

[75] The International Monetary Fund. World economic outlook database (available at http://www.imf.org/external/pubs/ft/weo/2013/01/weodata/index.aspx, accessed 22-sept-2014)).

[76] Sendi P, Günthard HF, Simcock M, Ledergerber B, Schüpbach J, et al (2007) Cost-effectiveness of genotypic antiretroviral resistance testing in HIV-infected patients with treatment failure. PLoS One 2: e173 10.1371/journal.pone.0000173 17245449 PMC1769464

