# Supplementary material for: Optimal Treatment Strategies in the Context of ‘Treatment for Prevention’ against HIV-1 in Resource-Poor Settings
Source: PLoS Comput Biol. 2015 Apr 30;11(4):e1004200. doi: 10.1371/journal.pcbi.1004200 (PMC4423987; doi:10.1371/journal.pcbi.1004200)
Supplement: S1 Text — (PDF) [file pcbi.1004200.s001.pdf]

Supplementary Text S1

Diagnostic-Guided Strategy: Closed-Loop Markov Control

Policy And Performance Criterion

For the **diagnostic-guided strategy**, we seek to find for each state x, an optimal time lapse $\tau(x)$ and an optimal action $a(x)$ that will be applied until the next diagnostic time point $t_{j+1} = t_j + \tau(x)$. The performance criterion $J(x, u)$ to be minimized is given by:

$$J(x,u) = \mathbb{E}_x^u \left(\sum_{j=0}^{\infty} e^{-\lambda t_j} \left(C\big(X_{t_j}, a(X_{t_j}), \tau(X_{t_j})\big) + e^{-\lambda \tau(X_{t_j})} k_{\text{dia}} \right) \right) \tag{1}$$

where $\mathbb{E}_x^u$ stands for the expectation value with respect to the measure determined by x and u.

Policy Iteration Algorithm

In order to compute the optimal policy for the Markov Control Process, dynamic programming techniques such as value iteration algorithm and policy iteration algorithm can be used. The value iteration algorithm iteratively calculates and improves the approximation of the value function until satisfactory convergence is achieved. In contrast, the policy iteration algorithm repetitively iterates over the set of policies for each state and improves them in each iteration until no further change occurs.

We implemented the policy iteration algorithm for our Markov control problem. In practice, the policy iteration algorithm has a polynomial run time [1, 2]. The algorithmic work-flow is as follows:

1. Start with one policy.
2. Calculate the utility based on the current policy.
3. Update the policy based on the policy formula.
4. Repeat Step 2 and 3 until the policy is stable.

Input

$T_{a,\tau}$; $C(x, a, \tau)$

Policy Iteration

u' \\\\ set an arbitrary policy

while $u \neq u'$

$u \leftarrow u'$

\\\\ compute the value function of u by solving the linear equations

$$J_u(x) = \left(C(x, a, \tau) + e^{-\lambda\tau} k_{\text{dia}} + \sum_{x' \in \mathcal{S}} e^{-\lambda\tau} \big(T_{a,\tau}[x', x] J_u(x') \big) \right). \tag{2}$$

for each $x \in \mathcal{S}$

\\\\ improve the policy

$$u'(x) = \underset{a,\tau}{\operatorname{argmin}} \left(C(x, a, \tau) + e^{-\lambda\tau} k_{\text{dia}} + \sum_{x' \in \mathcal{S}} e^{-\lambda\tau} \big(T_{a,\tau}[x', x] J_u(x') \big) \right).$$

end

end

The action space in our framework is given by $u(x) = \big(a(x), \tau(x)\big)$, thus has dimension $\mathcal{A} \times [0, \infty]$, where the set of possible diagnostic lag times τ needs to be discretized and limited in order to get a numerically feasible set. However, this is a justifiable step: Depending on the specific application there may be a lower- and upper examination lag time τ_{min} and τ_{max}, respectively. In our application, we set $\tau_{\text{min}} = 1$ days and $\tau_{\text{max}} = 5000$ days in order to numerically compute the optimal policy via the modified policy iteration algorithm presented above.

References

1. Ye Y (2010). The simplex and policy-iteration methods are strongly polynomial for the markov decision problem with a fixed discount rate.

2. Littman ML, Dean TL, Kaelbling LP (1995). On the complexity of solving markov decision problems.
