# Supplementary material for: Optimal Treatment Strategies in the Context of ‘Treatment for Prevention’ against HIV-1 in Resource-Poor Settings
Source: PLoS Comput Biol. 2015 Apr 30;11(4):e1004200. doi: 10.1371/journal.pcbi.1004200 (PMC4423987; doi:10.1371/journal.pcbi.1004200)
Supplement: S2 Text — (PDF) [file pcbi.1004200.s002.pdf]

Supplementary Text S2

Pro-active Strategy: Open-Loop Optimal Control

For computing the optimal **pro-active strategy**, one could naively forward propagate the initial probability vector p_0 by evaluating all controls (combinations of treatments), or backward propagate the terminal adjoint vector $\xi_{N_\mathcal{I}}$ through all possible controls to find the optimal control u^*. However, the size of the control space $\mathcal{U}$ depends on the number of actions available $|\mathcal{A}|$ at each interval and the number of intervals in which the physician *could* change the treatment, i.e $|\mathcal{U}| = |\mathcal{A}|^{N_\mathcal{I}}$. This implies that the memory and computational demands grow exponentially (in our case $|\mathcal{U}| = 2^{2500} >> 10^{625}$). Such demands render a naive approach impractical. Further, the two point boundary value nature of the problem makes it difficult to compute a solution by regular iteration techniques [1].

In order to numerically solve the problem, we introduce a dynamic programming technique that circumvents the necessity to consider all possible controls. We developed the algorithm for the *Bolza type* of performance criterion. The algorithm should be viewed as an extension of the algorithm introduced by Hernandez-Vargas [1], which was developed for a *Mayer type* of performance criterion (i.e., only terminal costs).

In the following we focus on a dynamic programming algorithm that backward propagates the adjoint vector. The naive approach requires to consider the backward-propagation of all possible adjoint vectors ξ_j at each interval j. If a test can be devised, such that at each interval j, some non-optimal adjoint vectors can be identified and discarded, then successive unnecessary computation of non-optimal adjoint vectors can be avoided by working on a search space that is $\ll |\mathcal{U}|$. The problem of identifying such non-optimal adjoint vectors can be casted as the problem to *identify and remove redundant constraints in a set of linear constraints.* For this type of problem, it is guaranteed that if a constraint associated with an adjoint vector is redundant, then the corresponding adjoint vector is non-optimal and can be removed from the set. For the identification of redundant constraints in linear programming, we used the algorithm introduced by *Caron et al.* [2,3]. Moreover, we explored ways to make the algorithm more efficient in identifying non-optimal adjoint vectors and suggest ways to parallelize the algorithm to further boost the speed. Details and the pseudo-code are given below.

Policy And Performance Criterion

For the **pro-active strategy**, we seek a sequence of $(N_\mathcal{I}+1)$ actions $(u_0, u_1, \cdots, u_{N_\mathcal{I}-1}, u_\infty)$ for a given initial probability vector p_0 which minimizes the following *Bolza type* performance criterion:

$$J(p_0, u) = \sum_{j=0}^{N_\mathcal{I}-1} q'_{u_j,j} \cdot p_j + q'_{u_\infty} \cdot p_{N_\mathcal{I}} \tag{1}$$

where $q_{u_\infty} \in \mathbb{R}_+^{|S|}$ and $q_{u_j,j} \in \mathbb{R}_+^{|S|}$ are the terminal and the running cost vector respectively. The summation of running cost denotes the expected discount cost over the time span $(0, N_\mathcal{I} \cdot \tau]$ and the terminal cost denotes the expected discount cost due to the fixed action u_∞ after the time $N_\mathcal{I} \cdot \tau$.

Computation Of Running and Terminal Costs

Next, we derive the equation for the computation of cost vector $q_{a,j}$. Let us look at the cost function C. Given some initial probability distribution p, action $a \in \mathcal{A}$ and a time span τ, the cost function is denoted by (see *main manuscript*)

$$C(p, a, \tau) = \sum_{x \in \mathcal{S}} p[x] \cdot C(x, a, \tau) \tag{2}$$

Let us define a cost vector $\mathcal{K}_a$ such that its xth component represents the cost produced by state x and action a per unit time as $\mathcal{K}_a[x] := c(x,a) = c_{\mathcal{S}}(x) + c_{\mathcal{A}}(a)$. The derivation of cost function is shown below

$$
\begin{aligned}
C(p,a,\tau) &= \mathcal{K}_a' \cdot \left(\int_0^\tau e^{-\lambda \cdot t} \cdot \mathbf{e}^{L_a \cdot t} dt \right) \cdot p \\
&= \mathcal{K}_a' \cdot \left(\int_0^\tau \mathbf{e}^{-\lambda \cdot t \cdot Id} \cdot \mathbf{e}^{L_a \cdot t} dt \right) \cdot p \\
&= \mathcal{K}_a' \cdot \left(\int_0^\tau \mathbf{e}^{(L_a - \lambda \cdot Id) \cdot t} dt \right) \cdot p \\
&= \mathcal{K}_a' \cdot (L_a - \lambda \cdot Id)^{-1} \cdot \left(\mathbf{e}^{(L_a - \lambda \cdot Id) \cdot \tau} - Id \right) \cdot p
\end{aligned}
\tag{3}
$$

The cost incurred in an interval can be written as

$$
q_{a,j}' \cdot p_j = e^{-\lambda t_j} \cdot C\big(p_j, a, \tau\big) \tag{4}
$$

where

$$
q_{a,j}' = e^{-\lambda \cdot t_j} \cdot \mathcal{K}_a' \cdot (L_a - \lambda \cdot Id)^{-1} \cdot \left(\mathbf{e}^{(L_a - \lambda \cdot Id) \cdot \tau} - Id \right),
$$

as derived above. In a similar manner, we can also deduce the cost vector q_{u_∞}.

Dynamic Programming Algorithm - Backward Propagation

Now, all parts constituting the optimal control problem in eqs. (29)-(34) (*main manuscript*) have been defined. Let us look more closely at the optimal control problem:

$$
\begin{aligned}
J^*(p_0, u^*) &= \min_{u \in \mathcal{U}} \left(\xi_m' \cdot p_m + \sum_{j=0}^{m-1} q_{u_j,j}' \cdot p_j \right) \\
\text{w.r.t} \quad p_{i+1} &= T_{u_i} \cdot p_i \qquad ; \; p_0 = p(0) \\
\xi_l' &= \xi_{l+1}' \cdot T_{u_l} + q_{u_l,l}' \quad ; \; \xi_{N_\mathcal{I}}' = q_{u_\infty}'
\end{aligned}
\tag{5}
$$

where $i = 0 \dots (m-1)$ and $l = (N_\mathcal{I} - 1) \dots m$ and boundary conditions are given by $p_0 = p(0)$ and $\xi_{N_\mathcal{I}} = q_{u_\infty}$.

Redundant Constraints In Linear Programming

For any given adjoint vector at the mth interval ξ_m, one can augment it with the result of the optimal control until the mth interval. And from the definition of optimal solution, Eq. 6 must hold

$$
\xi_m' \cdot p_m^* + r_{m-1}^* - J^*(p_0, u^*(p_0)) \geq 0 \tag{6}
$$

where

$$
r_{m-1}^* := \sum_{j=0}^{m-1} q_{u_j^*,j} \cdot p_j^*
$$

is the cost incurred due to optimal control $u^*(p_0)$ until the $m-1$ interval and $J^*(p_0, u^*(p_0))$ is the optimal cost for the given initial state vector p_0. Obviously, the equality sign is satisfied by the optimal adjoint vector ξ_m^*, whereas the remaining adjoint vectors satisfy the inequality.

Now, consider that the final adjoint vector is propagated backward and all possible adjoint vectors for the mth interval $(\xi_{m,1} \cdots \xi_{m,n})$ are computed. Here the vector $\xi_{m,k}$ represents the kth adjoint vectors

at the mth interval (i.e. the kth candidate control). For each adjoint vector, Eq. 6 must hold. Those set of equations can be expressed in matrix form as shown below:

$$\begin{bmatrix} \xi'_{m,1} & 1 & -1 \\ \xi'_{m,2} & 1 & -1 \\ \vdots & \vdots & \vdots \\ \vdots & \vdots & \vdots \\ \xi'_{m,n} & 1 & -1 \end{bmatrix} \cdot \begin{bmatrix} p^*_m \\ r^*_{m-1} \\ J^*(p_0, u^*(p_0)) \end{bmatrix} \geq \begin{bmatrix} 0 \\ 0 \\ \vdots \\ \vdots \\ 0 \end{bmatrix} \tag{7}$$

Given the values p^*_m, r^*_{m-1} and $J^*(p_0, u^*(p_0))$ are known, the optimal adjoint vector can be identified easily and remaining adjoint vectors can be discarded. Obviously, these values are not known beforehand. However, it is ensured that the vector

$$\begin{bmatrix} p^*_m \\ r^*_{m-1} \\ J^*(p_0, u^*(p_0)) \end{bmatrix} \tag{8}$$

is confined inside a *feasible region* defined by the following constraints:

$$\begin{bmatrix} \xi'_{m,1} & 1 & -1 \\ \xi'_{m,2} & 1 & -1 \\ \vdots & \vdots & \vdots \\ \vdots & \vdots & \vdots \\ \xi'_{m,n} & 1 & -1 \end{bmatrix} \cdot \begin{bmatrix} \rho \\ \pi \\ \zeta \end{bmatrix} \geq \begin{bmatrix} 0 \\ 0 \\ \vdots \\ \vdots \\ 0 \end{bmatrix} \tag{9}$$

Next, we will devise a test to discard non-optimal adjoint vectors using the knowledge that the vector Eq. 8 is confined in the *feasible region* defined by Eq. 9. Firstly, we will establish the following

Claim 1. *The adjoint vector $\xi_{m,1}$ at the mth interval that results in a redundant constraint in Eq. 9 cannot be an optimal adjoint vector at the mth interval.*

Proof : In order to identify whether a constraint is redundant or not, we apply the *linear programming method* for the identification of a redundant constraint, see i.e. [2, 3]. Without loss of generality, let us take the adjoint vector $\xi_{m,1}$ and the redundancy test can be expressed as the following linear programm:

$$\begin{aligned} \mu = \min_{\rho,\pi,\zeta} \; & \begin{bmatrix} \xi'_{m,1} & 1 & -1 \end{bmatrix} \cdot \begin{bmatrix} \rho \\ \pi \\ \zeta \end{bmatrix} \\ \text{w.r.t} \; & \begin{bmatrix} \xi'_{m,2} & 1 & -1 \\ \vdots & \vdots & \vdots \\ \vdots & \vdots & \vdots \\ \xi'_{m,n} & 1 & -1 \end{bmatrix} \cdot \begin{bmatrix} \rho \\ \pi \\ \zeta \end{bmatrix} \geq \begin{bmatrix} 0 \\ \vdots \\ \vdots \\ 0 \end{bmatrix} \\ & \sum_i \rho\,[i] = 1 \\ & p_{min} \leq \rho \leq p_{max} \\ & 0 \leq \pi \leq J_{max} \\ & J_{min} \leq \zeta \leq J_{max}, \end{aligned} \tag{10}$$

where p_{min},p_{max} and J_{min},J_{max} are lower and upper bounds for p^*_m and $J^*(p_0, u^*(p_0))$ respectively. If minimization yields $\mu > 0$, then the constraint associated with the adjoint vector $\xi_{m,1}$ is redundant and can be discarded (see Claim 1). Let the optimal solution for the linear programming Eq. 10 be the vector

$$\begin{bmatrix} \rho^{LP*} \\ \pi^{LP*} \\ \zeta^{LP*} \end{bmatrix} \tag{11}$$

If the constraint under consideration is redundant i.e., $\mu > 0$, then the following must hold

$$\begin{bmatrix} \xi'_{m,1} & 1 & -1 \end{bmatrix} \cdot \begin{bmatrix} p^*_m \\ r^*_{m-1} \\ J^*(p_0, u^*(p_0)) \end{bmatrix} \geq \begin{bmatrix} \xi m' & 1 & -1 \end{bmatrix} \cdot \begin{bmatrix} \rho^{LP*} \\ \pi^{LP*} \\ \zeta^{LP*} \end{bmatrix} = \mu > 0$$
$$\Rightarrow \xi'_{m,1} \cdot p^*_m + r^*_{m-1} - J^*(p_0, u^*(p_0)) > 0$$
$$\Rightarrow \xi'_{m,1} \cdot p^*_m + r^*_{m-1} > J^*(p_0, u^*(p_0))$$

thus the adjoint vector $\xi'_{m,1}$ cannot be optimal for the mth interval. □

The capability to recognize some non-optimal adjoint vectors at the mth interval motivates the following dynamic programming technique. One iterates over all adjoint vectors at the mth interval, performs the redundancy test (Eq. 10) for each adjoint vector and discard redundant ones, since it is guaranteed that they are non-optimal at the mth interval. Discarding such non-optimal vectors results in fewer adjoint vectors to be backward-propagated in the next interval $(\mathrm{m}\text{-}1)^{\mathrm{th}}$. At the $\mathrm{m}\text{-}1^{\mathrm{th}}$ interval, the process is repeated, and so on.

In each interval, the backward dynamic programming algorithm discards a set of non-optimal adjoint vectors and keeps a set of candidate adjoint vectors that contain the optimal adjoint vector (the optimal control). Instead of backward propagating all adjoint vectors in the mth interval to the $(\mathrm{m}\text{-}1)^{\mathrm{th}}$, the algorithm propagates only a smaller set of adjoint vectors (candidate set), thus prevents to explore *all* combinations.

Note, that it is not guaranteed that all non-optimal adjoint vectors for an interval will be identified as redundant constraints. However, the redundancy test can be improved by adding stricter bounds and constraints on variables ρ, π and ζ such that more non-optimal adjoint vectors are recognized. While adding bounds and constraints, it should be ensured that the resulting feasible region should still contain the optimal vector, see Eq. 8.

Additional Constraints And Bounds

Next, we will discuss the computation of additional constraints that will make the redundancy test more efficient. Following constraints can be added to the variable ρ.

1. Since the p^*_m is a probability vector, the sum of its components should add up to one and its component should be between 0 and 1. Thus, the constraints

$$\sum_{x \in \mathcal{S}} \rho\,[x] = 1 \tag{12}$$

and

$$0 \leq \rho\,[x] \leq 1 \tag{13}$$

can be added to the redundancy test.

2. We can further restrict the upper and lower bounds of p_m. Let us construct matrices T_{max} and T_{min} taking the component-wise maximum and minimum from all the matrices T_a as shown below

$$\begin{aligned} T_{max}[x,y] &= \max_{a \in \mathcal{A}} T_a\,[x,y] \\ T_{min}[x,y] &= \min_{a \in \mathcal{A}} T_a\,[x,y] \end{aligned} \tag{14}$$

Define vectors p_{DynLB} and p_{DynUB} such that

$$\begin{aligned} p_{DynLB} &:= T^m_{min} \cdot p_0 \\ p_{DynUB} &:= T^m_{max} \cdot p_0 \end{aligned} \tag{15}$$

then, any vector p_m should be bounded

$$p_{DynLB} \leq p_m \leq p_{DynUB} \tag{16}$$

These vectors can be computed for each interval m, as the algorithm proceeds. Note, that the matrices T_{min} and T_{max} are not stochastic anymore.

3. For a given action a at the interval m, the state equation is given by $p_{k+1} = T_a \cdot p_k$. Since the xth component of p_{k+1} and p_k are between 0 and 1 for all i, then any xth component is bounded as shown below

$$\min_y T_a[x,y] \leq p_{k+1}[x] \leq \max_y T_a[x,y] \tag{17}$$

Further irrespective of any action applied, the xth component of p is bounded as shown below

$$\min_{a\in\mathcal{A}} \min_y T_a[x,y] \leq p_{k+1}[x] \leq \max_{a\in\mathcal{A}} \max_y T_a[x,y] \tag{18}$$

Now let us define lower bound p_{StaLB} and upper bound p_{StaUB} vectors as below

$$\begin{aligned} p_{StaLB}\,[x] &:= \min_{a\in\mathcal{A}} \min_y T_a\,[x,y] \\ p_{StaUB}\,[x] &:= \max_{a\in\mathcal{A}} \max_y T_a\,[x,y] \end{aligned} \tag{19}$$

4. From the Eq. 13, Eq. 16, Eq. 18 and Eq. 19, the tighter upper and lower bound for p_m can be computed as follow

$$\begin{aligned} p_{min}[x] &:= \max(0, p_{StaLB}[x], p_{DynLB}[x]) \\ p_{max}[x] &:= \min(1, p_{StaUB}[x], p_{DynUB}[x]) \end{aligned} \tag{20}$$

such that for any state vector p_m at the interval mth, the bounds $p_{min} \leq p_m \leq p_{max}$ hold. Thus, following bounds for variable ρ can be added in the linear programming

$$p_{min} \leq \rho \leq p_{max} \tag{21}$$

For the bounds on the variable ζ, we can estimate the lower and upper bound of $J^*(p_0, u^*(p_0))$.

1. Since the optimal cost by definition is the minimum of all costs, a cost $J(p_0, u)$ due to any control $u \in \mathcal{U}$ can serve as a upper bound for the optimal cost $J^*(p_0, u^*(p_0))$. Instead of selecting any arbitrary control, selection of a constant control such that each $u_j = a$ has an advantage from the algorithmic perspective. As the backward algorithm proceeds, the upper bound can be updated to give a tighter bound. In the mth interval, any adjoint vector $\xi_{m,i}$ from the candidate sets can be propagated backward using $(u_0 = a, \cdots u_{m-1} = a)$ and from the definition of optimal control the following must hold

$$\xi'_{m,i} \cdot T_a^m \cdot p_0 + \sum_{k=0}^{m-1} q'_{u_k=a,k} \cdot T_a^k \cdot p_0 \geq J^*(p_0, u^*(p_0)) \tag{22}$$

We can iterate over all the possible actions and all candidate adjoint vectors to give us a tighter upper bound for the optimal cost as shown below

$$J_{max} := \min_a \min_i \; \xi'_{m,i} \cdot T_a^m \cdot p_0 + \sum_{k=0}^{m-1} q'_{u_k=a,k} \cdot T_a^k \cdot p_0 \geq J^*(p_0, u^*(p_0)) \tag{23}$$

2. The derivation of the lower bound for the optimal cost is shown below

$$\begin{aligned} J(p_0, u^*(p_0)) &= {\xi^*_m}' \cdot p^*_m + r^*_{m-1} \\ &= \left(\min_i \xi'_{m,i} \cdot p^*_m\right) + r^*_{m-1} \\ &\geq \left(\min_i \xi'_{m,i} \cdot p_{min}\right) + r^*_{m-1} \\ &\geq \left(\min_i \xi'_{m,i} \cdot p_{min}\right) =: J_{min} \end{aligned} \tag{24}$$

Thus for the variable ζ, the bound $J_{min} \leq \zeta \leq J_{max}$ can be added to the linear programming Eq. 10. Since the optimal cost until the (m-1)th interval r^*_{m-1} is smaller than the total optimal cost $J^*(p_0, u^*(p_0))$, we can add the bound $0 \leq \pi \leq J_{max}$ for the variable π.

Pseudo Codes

In order to explain the pseudo code for backward dynamic programming algorithm, we will introduce some notations. Given a matrix M, $M[j,]$ denotes the jth row of the matrix, whereas $M[\neg j,]$ denotes all rows of matrix M except the jth row. Let us define an operation $\oplus : \mathbb{R}^{n\times n} \oplus \mathbb{R}^n \mapsto \mathbb{R}^{n,n}$ which denotes the addition of a particular vector in $\mathbb{R}^n$ to each column of the matrix from $\mathbb{R}^{n,n}$. This is illustrated in the example below

$$\begin{bmatrix} \vdots & \vdots & \vdots & \vdots \\ a & b & \vdots & c \\ \vdots & \vdots & \vdots & \vdots \end{bmatrix}_{n\times n} \oplus \begin{bmatrix} \vdots \\ v \\ \vdots \end{bmatrix}_n = \begin{bmatrix} \vdots & \vdots & \vdots & \vdots \\ a+v & b+v & \vdots & c+v \\ \vdots & \vdots & \vdots & \vdots \end{bmatrix}_{n\times n}$$

The pseudo code for the backward dynamic programming is given below.

Input

p_0 ; q_{u_∞} ; T_a ; $q_{a,k}$ where $a = 1$ till N and $k = 1$ till T

Precomputation

$p_{StaLB} = \min_a \min_s T_a[s,]$; $p_{StaUB} = \max_a \max_s T_a[s,]$

for $a = 1$ till N \\ Iterate over actions

$\mathcal{P}_{a,0} = p_0$; $\mathcal{R}_{a,0} = 0$

for $j = 1$ till T \\ Iterate over intervals

$\mathcal{P}_{a,j} = T_a \cdot \mathcal{P}_{a,j}$

$\mathcal{R}_{a,j} = \mathcal{R}_{a,j} + q_{a,j} \cdot \mathcal{P}_{a,j}$

end

end

Backward Dynamic Programming Algorithm (BDA)

$\Theta_{N_\mathcal{I}} = q_{u_\infty}$ \\ Initialization

for $k = N_\mathcal{I} - 1$ till 0 \\ Backward Propagation

$\Theta_k = [T_1' \cdot \Theta_{k+1} \oplus q_{1,k}, T_2' \cdot \Theta_{k+1} \oplus q_{2,k}, \cdots T_N' \cdot \Theta_{k+1} \oplus q_{N,k}]$ \\Adjoint vectors

\\Updating the bounds for the state vector

$p_{DynLB} = T_{min}^k \cdot p_0$; $p_{DynUB} = T_{max}^k \cdot p_0$

for $s = 1$ Till $|\mathcal{S}|$

$p_{min}[s] = \max(0, p_{DynLB}[s], p_{StaLB}[s])$

$p_{max}[s] = \min(1, p_{DynUB}[s], p_{StaUB}[s])$

end

\\Updating the bounds for the optimal cost (25)

$J_{min} = \min_j \ \Theta_k[j,]' \cdot p_{min}$

$J_{max} = \min_a \min_j \ \Theta_k[j,]' \cdot \mathcal{P}_{a,k} + \mathcal{R}_{i,k}$

for all columns $\Theta_k[j,]$ \\ Parallelize the redundancy test

$$\mu_{k,j} = \min \ \begin{bmatrix} \Theta_k[j,]' & 1 & -1 \end{bmatrix} \cdot \begin{bmatrix} \rho \\ \pi \\ \zeta \end{bmatrix}$$

$$\text{w.r.t} \ \begin{bmatrix} \Theta_k[\neg j,]' & \overrightarrow{1} & -\overrightarrow{1} \end{bmatrix} \cdot \begin{bmatrix} \rho \\ \pi \\ \zeta \end{bmatrix} \geq \overrightarrow{0}$$

$$\sum_i \rho[i] = 1$$

$$p_{min} \leq \rho \leq p_{max}$$

$$0 \leq \pi \leq J_{max}$$

$$J_{min} \leq \zeta \leq J_{max}$$

if $\mu_{k,j} > 0$

Delete the column $\Theta_k[j,]$ from Θ_k

end

end

end

$J^* = \min \ \Theta_0' \cdot p_0$

Trace back the optimal control

Parallelization of Tests

The removal of redundant constraints does not change the feasible region. For a particular adjoint vector, its redundancy test is independent of redundancy test of other adjoint vectors at interval m. Instead of sequentially iterating over all k adjoint vectors at interval m, redundancy tests for adjoint vectors can be performed in parallel. The parallelization of tests can be used to boost the speed of the algorithm by exploiting the multi-core architecture of the modern computers.

References

1. Hernandez-Vargas E, Colaneri P, Middleton R, Blanchini F (2011) Discrete-time control for switched positive systems with application to mitigating viral escape. International Journal of Robust and Nonlinear Control 21: 1093–1111.

2. Caron R, McDonald J, Ponic C (1989) A degenerate extreme point strategy for the classification of linear constraints as redundant or necessary. Journal of Optimization Theory and Applications 62: 225–237.

3. Paulraj S, Sumathi P (2010) A comparative study of redundant constraints identification methods in linear programming problems. Mathematical Problems in Engineering 2010: 1-16.
