# Supplementary material for: Optimal Treatment Strategies in the Context of ‘Treatment for Prevention’ against HIV-1 in Resource-Poor Settings
Source: PLoS Comput Biol. 2015 Apr 30;11(4):e1004200. doi: 10.1371/journal.pcbi.1004200 (PMC4423987; doi:10.1371/journal.pcbi.1004200)
Supplement: S3 Text — (PDF) [file pcbi.1004200.s003.pdf]

Supplementary Text S3

Parameter-Sensitivity of Optimal Pro-active Strategy

Here, we analyze the sensitivity of the optimal **pro-active strategy** with respect to perturbations in cost parameters. It is realistic to assume that parameters related to costs (treatment, health, inflation, etc...) may change over time and may differ in distinct settings, unlike parameters related the viral dynamics (see Table 1, *Main Manuscript*).

Loss of productivity

In the *Main Manuscript*, we used values pL(h) = 0.4 and pL(m) = 0.1 [1] to quantify the loss of productivity related to viral copy number states h and m. We varied those values by factors of 2, 1, 1/2 and 1/4 and recomputed the optimal **pro-active strategy**, see Table S1 below. The optimal **pro-active strategy** remained identical in all cases. Naturally, a higher loss of productivity resulted in higher state costs and a lower loss of productivity resulted in lower state costs.

Table S1. Expected discounted cost of the optimal pro-active strategy for varying loss of productivity

Loss of productivity			Pro-active strategy			Optimal
pL(ℓ)	pL(m)	pL(h)	State cost	Treatment cost	Total cost	Strategy
0	0.2	0.8	86,239	2,771	89,010	a_1 for 15 days, then a_2
0*	0.1*	0.4*	81,048	2,771	83,819	a_1 for 15 days, then a_2
0	0.05	0.2	78,453	2,771	81,224	a_1 for 15 days, then a_2
0	0.025	0.1	77,156	2,771	79,927	a_1 for 15 days, then a_2

* Reference parameter set used in the *Main Manuscript*.

Treatment cost

In the *Main Manuscript*, the cost values used for the first- $c_{\mathcal{A}}(a_1)$ and second treatment line $c_{\mathcal{A}}(a_2)$ were 0.3 US$ and 1.08 US$, respectively [2]. Thus, the second treatment line is roughly 3.6 times more expensive than the first treatment line. We varied the cost of second treatment line in relation to the cost of the first treatment line by factors 1, 2, 3, 3.6 and 4. Table S2 shows the expected discounted costs of the optimal **pro-active strategy** for varying relative costs of the second treatment line. Despite the parameter perturbations, the optimal **pro-active strategy** remained identical (see Table S2), which shows that the control is relatively insensitive to changes in treatment costs. Naturally, the state costs remain identical and the treatment- and total costs increase with increasing costs of the second treatment line.

Inflation

We used a performance criterion that quantifies the expected costs over an infinite time horizon (see eq. (22)-(23), *Main Manuscript*). By introducing a discount factor λ, costs arising immediately are weighted more than uncertain future costs. Moreover, the discount factor guarantees convergence of the performance criterion. In our computations, we used the annual inflation in South Africa (5.4% [3]) in order to compute λ. We varied the inflation rate from 3 to 8%. The resulting costs are stated in Table S3. The optimal **pro-active strategy** remained unchanged. With increasing inflation rates, the expected discounted costs decrease.

Table S2. Expected discounted costs of the optimal pro-active strategy for varying relative treatment costs $c_{\mathcal{A}}(a_1) : c_{\mathcal{A}}(a_2)$

ratio $c_{\mathcal{A}}(a_1)$:$c_{\mathcal{A}}(a_2)$	Pro-active strategy State cost	Treatment cost	Total cost	Optimal strategy
1:1	81,048	773	81,821	a_1 for 15 days, then a_2
1:2	81,048	1,542	82,590	a_1 for 15 days, then a_2
1:3	81,048	2,310	83,358	a_1 for 15 days, then a_2
1:3.6*	81,048	2,771	83,819	a_1 for 15 days, then a_2
1:4	81,048	3,078	84,126	a_1 for 15 days, then a_2

* Reference parameter set used in the *Main Manuscript.*

Table S3. Expected discounted costs of the optimal pro-active strategy for different inflation rates

Inflation rate per annum λ [%]	Pro-active strategy State cost	Treatment cost	Total cost	Optimal strategy
3	172,037	3,269	175,306	a_1 for 15 days, then a_2
4	120,081	3,042	123,124	a_1 for 15 days, then a_2
5.4*	81,048	2,771	83,819	a_1 for 15 days, then a_2
6	70,263	2,511	72,932	a_1 for 15 days, then a_2
7	56,750	2,511	59,261	a_1 for 15 days, then a_2
8	46,948	2,371	49,319	a_1 for 15 days, then a_2

* Reference parameter used in the *Main Manuscript.*

References

1. Sendi P, Günthard HF, Simcock M, Ledergerber B, Schüpbach J, et al. (2007) Cost-effectiveness of genotypic antiretroviral resistance testing in HIV-infected patients with treatment failure. PLoS One 2: e173.

2. The Clinton Health Access Initiative (2011). Antiretroviral (ARV) ceiling price list (available at `http://www.clintonfoundation.org`, accessed 22-sept-2014).

3. The International Monetary Fund. World economic outlook database (available at `http://www.imf.org/external/pubs/ft/weo/2013/01/weodata/index.aspx`, accessed 22-sept-2014)).
