# Supplementary material for: Optimal Treatment Strategies in the Context of ‘Treatment for Prevention’ against HIV-1 in Resource-Poor Settings
Source: PLoS Comput Biol. 2015 Apr 30;11(4):e1004200. doi: 10.1371/journal.pcbi.1004200 (PMC4423987; doi:10.1371/journal.pcbi.1004200)
Supplement: S4 Text — (PDF) [file pcbi.1004200.s004.pdf]

Supplementary Text S4

Ongoing Treatment vs. Optimal Pro-active Strategy

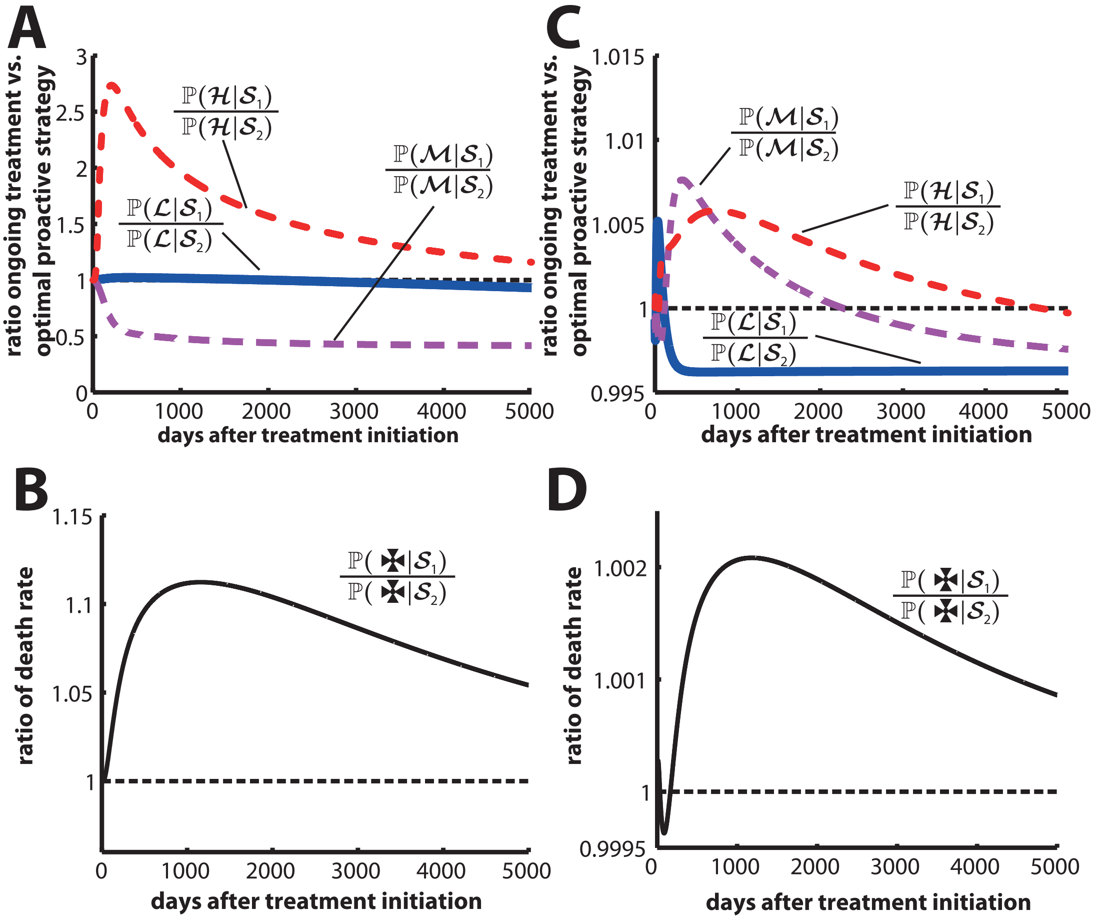

Figure S1. Ongoing treatment in comparison to the optimal pro-active strategy. Left panels: Dynamics of a constant treatment a_1 in relation to the optimal **pro-active strategy**. **Right panels:** Dynamics of a constant treatment a_2 in relation to the optimal **pro-active strategy**. In **A & C** solid blue lines represent the probability ratio of states $\mathcal{L}$ for ongoing treatment with a_1 or a_2 (strategy S_1) in relation to the optimal **pro-active strategy** (strategy S_2) i.e., $(P(\mathcal{L}|S_1)/(P(\mathcal{L}|S_2)))$. Magenta- and red dashed lines show the ratios for the sets $\mathcal{M}$ and $\mathcal{H}$. In **B & D**, the solid black lines represent the ratio of death probabilities. The thin black horizontal lines represents the line of unity in all figures.

The model predicted HIV-dynamics, if no treatment switches were applied, relative to the dynamics under the optimal **pro-active strategy** are shown in Fig. S1A-D. Fig. S1A&C show the dynamics for the sets of states $\mathcal{L}$, $\mathcal{M}$ and $\mathcal{H}$. The set $\mathcal{L}$ denotes the set of states for which condition $n_C(M) \leq \ell$ for all possible virus mutants M holds (i.e. undetectable total virus load), $\mathcal{H}$ refers to all states for which for at least one viral strain M, $n_C(M) > m$ (high virus loads). The remaining viral states belong to

$\mathcal{M}$. Fig. S1B&D show the relative death probabilities for a constant treatment in relation to an optimal **pro-active strategy**.

Figure S1A shows that the relative probability of set $\mathcal{H}$ (red dashed line; *h*igh viral loads) is higher if treatment with a_1 is maintained in comparison to the optimal **pro-active strategy**. After roughly 200 day, the probability of set $\mathcal{H}$ becomes ≈2.7 times the probability of set $\mathcal{H}$ in the **pro-active strategy** . Application of a_1 without treatment change also results in an increased death probability (Figure S1B) with a peak after about 1000 days. As a consequence of patient death, the relative probability of being in set $\mathcal{H}$ declines over time in Fig. S1A.

The comparison between ongoing treatment with a_2 and the optimal **pro-active strategy** is depicted in Figure S1C-D. For the first few weeks, the probability of set $\mathcal{L}$ is higher with a_2 and the death probability is lower than with the optimal **pro-active strategy**. However, the performance of the ongoing treatment with a_2 deteriorates and in the long term the optimal **pro-active strategy** outperforms ongoing treatment with a_2. After roughly 200 days, the death rate is higher and probability of state $\mathcal{L}$ is lower for ongoing treatment with a_2 than with the optimal **pro-active strategy**. Therefore, the ongoing treatment with a_2 may be effective in the short term, but in the long term the emergence of drug resistant strains R2 may undermine its short-term efficacy. The optimal pro-active strategy delays the emergence of strains resistant to treatment a_2 and remains more effective in a long run.
