# Supplementary material for: Optimal Treatment Strategies in the Context of ‘Treatment for Prevention’ against HIV-1 in Resource-Poor Settings
Source: PLoS Comput Biol. 2015 Apr 30;11(4):e1004200. doi: 10.1371/journal.pcbi.1004200 (PMC4423987; doi:10.1371/journal.pcbi.1004200)
Supplement: S5 Text — (PDF) [file pcbi.1004200.s005.pdf]

Supplementary Text S5

Determination of Incidence Rates for Different HIV Levels

A growing body of evidence suggests a direct association between *plasma* HIV-1 levels (viral load) and the HIV transmission risk during intercourse [1–3]. The reason may be that the primary determinant of sexual transmission, *genital* HIV-1 levels, correlate with *plasma* HIV-1 levels [4–7], although some exceptions have been reported [8, 9].

Data

The meta-analysis conducted by *Attia et al* [10] systematically reviews 11 clinical studies on discordant heterosexual couples from sub-saharan Africa. The primary focus of the analysis was to differentiate the overall incidence rate between the groups receiving anti-retroviral treatment (ART) and the non-treated individuals, based on differences in plasma HIV-1 levels. Incidence rates (and their confidence intervals) from [10] for different HIV-1 levels are shown in Fig. 1 (black dots and error bars).

Relation between viral load and transmission risk

In order to describe the relation between plasma HIV levels and HIV incidence rates, *Hughes et al.* [11] suggested to approximate transmission risks by the square root of the plasma viral load. In a similar vein, we fitted incidence rates per 100 person years as a square root function of plasma viral load:

$$\mathbb{IR}(v) = b \cdot \sqrt{v} \tag{1}$$

where $\mathbb{IR}$ is the incidence rate, v is the total viral load (plasma HIV-1 RNA level) and b is a parameter to be estimated.

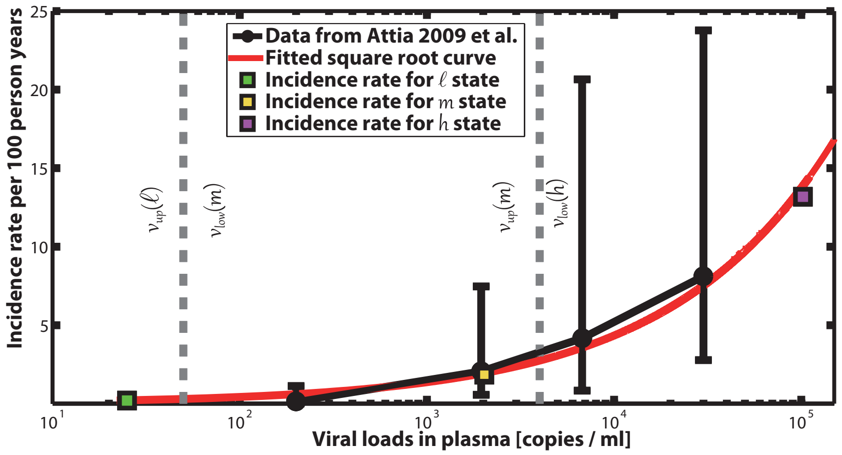

Figure 1. Incidence rates for different HIV levels in blood plama: The black dots and vertical bars represent mean and the 95% confidence interval of the incidence rates reported in *Attia et al* [10]. The red line represent the fit of eq. (1) to the data. The green, yellow and purple squares represent the average incidence rates per 100 person years for lumped states ℓ, m and h as shown in eqs. (2)-(3)

The red line in Figure 1 represents the fitted square root function, which is in good agreement with the data. Each 1 $\log_{10}$ increase in plasma viral load increased the incidence rate by 3.16 fold, which is within the range of published values (range: 1.79–3.26 [2, 12, 13]).

Transmission risk for lumped viral states

For a lumped copy number states $n_C \in \{\ell, m, h\}$ of our HIV-model with $v_{low}(n_C)$ and $v_{up}(n_C)$ as lower and upper viral load range as defined in the *main manuscript*, the incidence rate was estimated to be

$$\mathbb{IR}(n_C) = \frac{\int_{v_{low}(n_C)}^{v_{up}(n_C)} \mathbb{IR}(v)\, dv}{v_{up}(n_C) - v_{low}(n_C)}. \tag{2}$$

The incidence rates for lumped states ℓ, m and h were estimated to be 0.20, 1.85 and 13.18 per 100 person years respectively and are illustrated by green-, yellow- and purple squares in Figure 1.
Since the transmission risk is related to the total virus load, for each state x of the HIV model, the incidence rate is given by

$$\mathbb{IR}(x) = \max_{n_C}\ \mathbb{IR}(n_C). \tag{3}$$

References

1. Fideli US, Allen SA, Musonda R, Trask S, Hahn BH, et al. (2001) Virologic and immunologic determinants of heterosexual transmission of human immunodeficiency virus type 1 in Africa. AIDS Res Hum Retroviruses 17: 901–910.

2. Quinn TC, Wawer MJ, Sewankambo N, Serwadda D, Li C, et al. (2000) Viral load and heterosexual transmission of human immunodeficiency virus type 1. Rakai Project Study Group. N Engl J Med 342: 921–929.

3. Lingappa JR, Hughes JP, Wang RS, Baeten JM, Celum C, et al. (2010) Estimating the impact of plasma HIV-1 RNA reductions on heterosexual HIV-1 transmission risk. PLoS One 5: e12598.

4. Goulston C, McFarland W, Katzenstein D (1998) Human immunodeficiency virus type 1 RNA shedding in the female genital tract. J Infect Dis 177: 1100–1103.

5. Iversen AK, Larsen AR, Jensen T, Fugger L, Balslev U, et al. (1998) Distinct determinants of human immunodeficiency virus type 1 RNA and DNA loads in vaginal and cervical secretions. J Infect Dis 177: 1214–1220.

6. Hart CE, Lennox JL, Pratt-Palmore M, Wright TC, Schinazi RF, et al. (1999) Correlation of human immunodeficiency virus type 1 RNA levels in blood and the female genital tract. J Infect Dis 179: 871–882.

7. Kovacs A, Wasserman SS, Burns D, Wright DJ, Cohn J, et al. (2001) Determinants of HIV-1 shedding in the genital tract of women. Lancet 358: 1593–1601.

8. Fiore JR, Suligoi B, Saracino A, Di Stefano M, Bugarini R, et al. (2003) Correlates of HIV-1 shedding in cervicovaginal secretions and effects of antiretroviral therapies. AIDS 17: 2169–2176.

9. Lorello G, la Porte C, Pilon R, Zhang G, Karnauchow T, et al. (2009) Discordance in HIV-1 viral loads and antiretroviral drug concentrations comparing semen and blood plasma. HIV Med 10: 548–554.

10. Attia S, Egger M, Müller M, Zwahlen M, Low N (2009) Sexual transmission of HIV according to viral load and antiretroviral therapy: systematic review and meta-analysis. AIDS 23: 1397–1404.

11. Hughes JP, Baeten JM, Lingappa JR, Magaret AS, Wald A, et al. (2012) Determinants of per-coital-act HIV-1 infectivity among african HIV-1-serodiscordant couples. J Infect Dis 205: 358–365.

12. Nagelkerke NJD, Arora P, Jha P, Williams B, McKinnon L, et al. (2014) The rise and fall of HIV in high-prevalence countries: a challenge for mathematical modeling. PLoS Comput Biol 10: e1003459.

13. Baeten JM, Kahle E, Lingappa JR, Coombs RW, Delany-Moretlwe S, et al. (2011) Genital HIV-1 RNA predicts risk of heterosexual HIV-1 transmission. Sci Transl Med 3: 77ra29.
